# Severe pulmonary disease in an adult primary ciliary dyskinesia population in Brazil

**DOI:** 10.1038/s41598-019-45017-1

**Published:** 2019-06-18

**Authors:** Mary Anne Kowal Olm, Fernando Augusto Lima Marson, Rodrigo Abensur Athanazio, Naomi Kondo Nakagawa, Mariangela Macchione, Niki Tomas Loges, Heymut Omran, Samia Zahi Rached, Carmen Sílvia Bertuzzo, Rafael Stelmach, Paulo Hilário Nascimento Saldiva, José Dirceu Ribeiro, Marcus Herbert Jones, Thais Mauad

**Affiliations:** 1 Department of Pathology, São Paulo University Medical School, São Paulo, SP 01246-903 Brazil; 2 Department of Medical Genetics and Genomic Medicine, Faculty of Medical Science, University of Campinas, Campinas, SP 13083-887 Brazil; 3 Pulmonary Division, Heart Institute (InCor), Hospital das Clínicas da Faculdade de Medicina da Universidade de São Paulo, São Paulo, SP 05403-000 Brazil; 4 Department of Pediatrics and General Pediatrics, Muenster University Hospital, Muenster, 48149 Germany; 5 Department of Pediatrics, Faculty of Medical Science, University of Campinas, Campinas, SP 13083-887 Brazil; 6 Department of Pediatrics, Pontifical Catholic University of Rio Grande do Sul, Porto Alegre, RS 90610-000 Brazil

**Keywords:** Respiratory tract diseases, Molecular medicine

## Abstract

Primary Ciliary Dyskinesia (PCD) is underdiagnosed in Brazil. We enrolled patients from an adult service of Bronchiectasis over a two-year period in a cross-sectional study. The inclusion criteria were laterality disorders (LD), cough with recurrent infections and the exclusion of other causes of bronchiectasis. Patients underwent at least two of the following tests: nasal nitric oxide, ciliary movement and analysis of ciliary immunofluorescence, and genetic tests (31 PCD genes + *CFTR* gene). The clinical characterization included the PICADAR and bronchiectasis scores, pulmonary function, chronic *Pseudomonas aeruginosa* (cPA) colonization, exhaled breath condensate (EBC) and mucus rheology (MR). Forty-nine of the 500 patients were diagnosed with definite (42/49), probable (5/49), and clinical (2/49) PCD. Twenty-four patients (24/47) presented bi-allelic pathogenic variants in a total of 31 screened PCD genes. A PICADAR score > 5 was found in 37/49 patients, consanguinity in 27/49, LD in 28/49, and eight PCD sibling groups. FACED diagnosed 23/49 patients with moderate or severe bronchiectasis; FEV_1_ ≤ 50% in 25/49 patients, eight patients had undergone lung transplantation, four had been lobectomized and cPA+ was determined in 20/49. The EBC and MR were altered in all patients. This adult PCD population was characterized by consanguinity, severe lung impairment, genetic variability, altered EBC and MR.

## Introduction

Motile ciliopathies are characterized by their generation of abnormal fluid flow or movement within fluids, which compromises mucus clearance and results in chronic airway disease^1^. Amongst motile ciliopathies, Primary Ciliary Dyskinesia (PCD, OMIM: #244400) is a genetically heterogeneous recessive disorder that results in neonatal respiratory distress, chronic oto-sino-pulmonary diseases, male infertility and organ laterality defects in ~50% of cases^2^. The global incidence of PCD is estimated at one case per 10,000 to 20,000 births. An international registry including 18 countries has recently gathered data on 3,013 patients diagnosed with PCD^3^.

According to the PCD foundation consensus^4^, the diagnosis of PCD includes two major clinical criteria and at least one of the following altered tests: a low nasal nitric oxide (nNO) production rate on two occasions, diagnostic ciliary ultrastructure with Transmission Electron Microscopy (TEM), bi-allelic gene mutations in one PCD-associated gene^5^ and wave abnormalities on high-speed video microscopy analysis (HVMA)^4^. In addition, some services have used High-Resolution Immunofluorescence Microscopy (IFM) to confirm ultrastructure data^6^.

Considering the classification of the international PCD register, PCD patients can be separated into three groups: definitive diagnosis of PCD, established by identifying hallmark TEM findings and/or biallelic PCD mutation, probable PCD (patients with abnormal video movement and/or low nNO), and clinical PCD (negative or ambiguous tests but a strong clinical characteristic)^3^. However, the genetic tests for variants that determine alterations of ultrastructure and transport proteins for the diagnosis of PCD are not universally available^7^.

Despite the extensive number of patients who have been diagnosed with PCD in Europe^8^ and the United States^9^, in many countries, PCD diagnosis is still not performed, mainly due to the lack of resources. Moreover, few studies address the clinical characterization of PCDs in adults^10,11^, especially in terms of disease severity and the relation to severe pulmonary commitment^12^. In Brazil, there are no reference centres for PCD screening, diagnosis and management. Therefore, the prevalence of PCD is unknown in this country, and there are few data that characterize PCD patients^13^.

In this study, we aimed to identify, diagnose and fully characterize a group of adult PCD patients who were followed up at a Bronchiectasis Outpatient Service at a large tertiary care complex in São Paulo, Brazil.

## Results

### Demographics and clinical characteristics

Of the 500 patients registered at the Bronchiectasis Outpatient Service over a two-year period, 55 fulfilled the eligibility criteria for PCD assessment, and 49 were diagnosed with PCD. The six patients excluded presented normal nNO and ultrastructure and no laterality disorders. The characteristics of these patients are shown in the Supplementary File (Table S1).

The mean ± SD age of the patients was 41.6 ± 12.9 y, ranging from 21 to 77 y (28 M:21 F). The mean ± SD BMI was 23.8 ± 3.6 kg/m^2^ (ranging from 16.8 to 32.8 kg/m^2^). Furthermore, 45/49 (92%) of the patients were Caucasians and four patients were Afro descendants. Consanguinity was present in 27/49 (55%) of the patients, and there were eight sibling groups in this population (n = 18). The place of birth of the patients was divided between the southeast and northeast regions of the country, but consanguinity was more frequent in patients from the northeast part of the country (31%). Relatives with similar respiratory commitment were described in 28/49 (57%) patients.

The FACED scores ranged from mild in 20/49 patients (41%) to moderate in 14/49 patients (29%) to severe in 7/49 patients (14%). Chronic *P. aeruginosa* colonization (cPA) was found in 20/49 (41%) patients. The mean ± SD FEV_1_ in PCD patients was 49.2 ± 19.8 L/s, and 25 patients had FEV_1 < _50% predicted. Eight patients (16%) had previously undergone a lung transplant, and 4/49 (8%) had been lobectomized. A single patient (2%) was oxygen-dependent (for individual data, see Table 1).

**Table 1:** Clinical and demographic data from patients with phenotypes compatible with primary ciliary dyskinesia.

Patient	Age (y)	Sex	Race	Place of birth	Consanguinity	Relatives with same symptoms	BMI	FEV_1_ (%)/FCV (%)	FACED	cPa	Altered spermiogram	Lung surgery/oxygen dependent
Br-1	49	F	C	SE	No	1	25.5	33/41	6	+	NA (W)	LL
Br-2	39	M	C	NE	No	−	26.5	45/61	5	+	+	−
Br-3	63	M	C	SE	Yes	−	28.0	57/72	5	−	+	−
Br-4	22	M	C	SE	No	−	20.3	64/103	1	−	−	−
Br-5	56	F	C	SE	Yes	2	31.1	63/79	1	−	NA (W)	−
Br-6	30	M	AD	SE	Yes	−	21.1	62/82	1	−	+	−
Br-7	32	M	C	NE	Yes	−	22.2	60/54	NA-LT	+	+	TL
Br-8	35	M	C	NE	Yes	−	18.4	59/71	1	+	+	−
Br-9	54	F	AD	SE	No	−	30.0	32/39	4	−	NA (W)	−
Br-10	36	F	AD	NE	No	−	25.3	33/47	3	−	NA (W)	−
Br-11	25	M	C	NE	Yes	1	21.3	19/36	3	−	−	LL
Br-12	77	M	C	SE	Yes	−	24.1	28/41	3	−	Natural children	−
Br-13	57	M	C	NE	Yes	1	25.0	29/48	3	−	−	−
Br-14	33	F	C	SE	No	−	28.8	82/71	NA-LT	+	NA (W)	TL
Br-15	39	M	C	NE	No	2	23.3	67/79	1	−	+	−
Br-16	41	F	C	SE	No	−	28.1	70/83	2	−	NA (W)	−
Br-17	55	F	C	SE	No	−	26.4	54/66	4	+	NA (W)	−
Br-18	47	F	C	NE	No	−	23.0	47/64	2	+	NA (W)	LL
Br-19	45	M	C	NE	Yes	2	30.0	70/75	1	−	+	−
Br-20	37	M	C	NE	Yes	2	24.0	42/54	2	−	+	−
Br-21	23	F	C	NE	No	−	23.5	68/73	NA-LT	+	NA (W)	TL
Br-22	40	M	C	SE	Yes	1	21.4	31/50	3	−	+	−
Br-23	21	F	C	SE	No	1	25.7	56/81	3	+	NA (W)	−
Br-24	53	M	C	SE	No	−	20.1	18/32 (pT)	NA-LT	+	+	TL-
Br-25	41	M	C	NE	Yes	1	20.4	90/82	NA-LT	−	+	TL
Br-26	46	M	C	SE	Yes	3	17.6	29/53	1	−	+	−
Br-27	50	F	C	SE	Yes	3	23.8	36/53	3	−	NA (W)	−
Br-28	46	M	C	NE	Yes	1	23.0	34/49	6	+	+	Oxd
Br-29	22	F	C	SE	No	1	22.7	55/79	1	−	NA (W)	−
Br-30	52	F	C	SE	Yes	2	21.3	35/59	2	−	NA (W)	−
Br-31	59	F	W	SE	Yes	2	23.0	63/78	2	−	NA (W)	−
Br-32	56	M	AD	NE	Yes	1	23.2	39/61	5	+	+	−
Br-33	39	F	C	SE	No	−	24.3	64/79	2	−	NA (W)	−
Br-34	57	M	C	NE	Yes	1	24.8	32/54	2	−	−	−
Br-35	34	F	C	NE	Yes	1	20.0	28/44	5	+	NA (W)	−
Br-36	20	M	C	SE	No	−	21.6	67/74	2	+	−	−
Br-37	58	M	C	S	No	1	26.1	21/38 (pT)	NA-LT	+	+	TL
Br-38	39	M	C	NE	No	−	16.8	23/56 (pT)	NA-LT	−	+	TL
Br-39	25	F	C	NE	Yes	1	20.9	67/54	2	−	NA (W)	−
Br-40	47	M	C	SE	Yes	1	30.8	59/72	2	−	+	−
Br-41	54	M	C	SE	Yes	1	25.0	38/47	3	−	+	−
Br-42	48	F	C	NE	Yes	1	17.9	38/54	5	+	NA (W)	−
Br-43	24	M	C	SE	No	1	18.8	43/45	3	−	+	−
Br-44	33	F	C	NE	Yes	2	21.6	75/83	4	+	NA (W)	−
Br-45	33	M	C	NE	Yes	3	25.5	70/90	3	+	+	−
Br-46	27	M	C	SE	Yes	−	25.3	26/36 (pT)	NA-LT	+	+	TL
Br-47	49	F	C	NE	No	−	22.4	104/108	2	+	NA (W)	LL
Br-48	43	M	C	NE	No	1	32.8	37/60	3	−	Natural children	−
Br-49	27	F	C	SE	No	1	26.9	−	0	−	NA (W)	−

Legend: y, years; M, male; F, female; C, Caucasian; AD, afro-descendent; SE, from southeast Brazil; NE, from northeast Brazil; S, from south Brazil; FEV_1_, forced expiratory volume in the first second of the FVC; FVC, forced vital capacity; BMI, body index mass; pT, pre-transplant; NA, not applicable; TL, transplanted lung; LL, lobectomized lung; Oxd, Oxygen dependent; cPa, chronic *Pseudomonas aeruginosa* colonization; +, presence of colonization in cPA column; −, absence of colonization in cPA column; W, Woman; FACED, F – FEV_1_, A – Age, C – Chronic colonization, E – Extension, D – Dyspnea.

Table 2 presents the signs and symptoms related to these PCD patients.

**Table 2:** Clinical characteristics of the PCD patients.

Signs and Symptoms	Number of patients N = 49	%
Birth condition		
At term birth	33	67.3
Neonatal respiratory distress	16	32.7
Laterality disorder		
*Situs inversus*	23	46.9
Dextrocardia	3	6.1
Congenital heart disease	1	2.0
Polysplenia	1	2.0
Upper respiratory disease		
Chronic rhinitis	49	100.0
Chronic sinusitis	46	93.9
Chronic otitis	25	51.0
Hearing loss	37	75.5
Lower respiratory disease		
Chronic wet cough	49	100.0
Bronchiectasis	49	100.0
Recurrent pneumonia	26	53.1
Hemoptysis	14	28.6
Infertility		
Men	21	42.9
Women	9	18.4
Other		
Oxygen dependence	1	2.0
Previous lobectomy	4	8.2
Lung transplantion	8	16.3
Gastroesophageal reflux disease	17	34.7
Body Mass Index < 20	5	10.2
FEV_1 _< 50 (not transplanted)	24	49.0
Chronic *P. aeruginosa* colonization	20	40.8

Legend: FEV_1_- Forced Vital Capacity; P.- Pseudomonas.

### Diagnostic assessment

The PICADAR scores ranged from three to 14 points, with 37/49 (76%) patients with scores higher than five. Laterality disorders were present in 28/49 (58%) patients: 23/49 (47%) had s*itus inversus*, 3/49 (6%) had dextrocardia, 1/49 (2%) had congenital heart disease, and 1/49 (2%) had polysplenia (Table 3).

**Table 3:** Results from the tools used to determine the diagnosis of patients with phenotypes compatible with primary ciliary dyskinesia (PCD).

Patient	PICADAR/ Laterality disorder/Cg*/sibling	nNO (nL/min)	CBF/CBP	Cilia US (TEM)	Cilia IF altered Results (OBr code)	Genetic compatible with PCD diagnosis	Diagnosis
Br-1	4	8.9	Static	OIDA	DNAH5 (OBr31)	—	PCD
Br-2**	4	27.6	2.5 - Circle	ACP	RSPH9 (OBr35)	—	PCD
Br-3	12*, SI	5.7	Static	ODA	rs	*DNAI2* ^a,d^	PCD
Br-4	6*	9.6	Red ampl	MTD + IDA	CCDC39 (OBr9)	—	PCD
Br-5	10, SI, s1	14.1	1.7 - Static	ODA	DNAH5 (OBr32)	—	PCD
Br-6 π	3*	4.2	1.7 - Red ampl	MTD + IDA	CCDC39 (OBr19)	*RPGR*(X-chrom)	PCD
Br-7 ^£^	8*, SI	6.5	1.7- Red ampl	MTD + IDA	CCDC39 (OBr30)	*CCDC40* ^a,d^	PCD
Br-8	8	14.4	1.7- Red ampl	MTD + IDA	nd	*CCDC40* ^a,d^	PCD
Br-9	4	15.5	Static	OIDA	rs	—	PCD
Br-10	9*, SI	2.1	Altered	MTD + IDA	CCDC39 (OBr17)	*CCDC39* ^a,d^	PCD
Br-11	9*, SI, s2	7.5	1.7 - Red amp	MTD + IDA	CCDC39 (OBr10)	*CCDC40* ^a,d^	PCD
Br-12	7*, SI	18.3	Static	ODA	DNAH5 (OBr3)	*CCDC151* ^a,d^	PCD
Br-13	8*, SI, s3	7.4	Static	MTD + IDA	CCDC39 (OBr5)	—	PCD
Br-14	8, Ps	10.1	Static	OIDA	DNAH5 (OBr8)	*DNAAF3* ^a,d^	PCD
Br-15	8*, SI, s4	9.6	Altered	OIDA	DNAH5 (OBr21)	—	PCD
Br-16	12*, SI	18.6	3.3 - Red ampl	MTD + IDA	CCDC39 (OBr1)	*CCDC40* ^a,d^	PCD
Br-17	4	21.8	1.7 - Altered	ACP	GAS8 (OBr29)	*RSPH1* ^a,d^	PCD
Br-18	10*, CHD	17.1	Altered	ODA	DNAH5 (OBr6)	—	PCD
Br-19	4*, s4	7.5	1.7 - Altered	OIDA	nd	—	PCD
Br-20***	3*, s4	10.1	Static	OIDA	nd	*DNAH11* ^c,d^	PCD
Br-21	7*, SI	8.1	Static	ODA	DNAH5 (OBr12)	*DNAH5* ^a,d^	PCD
Br-22	9*, SI	5.9	Static	OIDA	nd	*DNAAF*3^c,d^	PCD
Br-23	8, s5	16.2	5 - Circle	ACP + T	nd	*RSPH1* ^a,d^	PCD
Br-24	8, SI	9.3	Altered	ODA	na	*DNAH5* ^a,d^	PCD
Br-25	6*, s2	nd	nd	MTD + IDA	nd	*CCDC40* ^a,d^	PCD
Br-26	8*, SI, s6	nd	nd	ODA	nd	*DNAH5* ^a,d^	PCD
Br-27	8*, SI, s6	nd	nd	ODA	nd	*DNAH5* ^a,d^	PCD
Br-28	6*, SI, s7	nd	nd	MTD + IDA	nd	*CCDC40* ^a,d^	PCD
Br-29	4, s5	nd	nd	ACP + T	nd	*RSPH1* ^a,d^	PCD
Br-30**	10, SI, s1	nd	nd	ODA	nd	—	PCD
Br-31**	10, SI, s1	nd	nd	ODA	nd	—	PCD
Br-32	8*, SI, s7	nd	nd	MTD + IDA	nd	*CCDC40* ^a,d^	PCD
Br-33	8, SI	nd	nd	ODA	nd	—	PCD
Br-34**	5*, s3	nd	nd	MTD + IDA	nd	—	PCD
Br-35	8, SI	19.8	nd	MTD + IDA	CCDC39 (OBr27)	*CCDC40* ^a,d^	PCD
Br-36	7	4.2	nd	ODA	DNAH5 (OBr23)	Not consent	PCD
Br-37	4	34.2	3.3-Circle	ACP + T	na	—	PCD
Br-38	4	10.4	Altered	MTD + IDA	nd	Not consent	PCD
Br-39	10*, SI	nd	nd	OIDA	nd	*DYX1C1-CCPG1* ^c,d^	PCD
Br-40	10*, s8	46.5	5.0-Altered	Inconcl.	nd	*DYX1C1-CCGP1* ^b,d^	PCD
Br-41	7, * SI, s8	nd	nd	Inconcl.	nd	*DYX1C1-CCGP1* ^b,d^	PCD
Br-42**	6*	7.1	3.3-Circle	ACP	nd	—	PCD
Br-43	13, Dx	39.5	Altered	Normal	DNAH11 (OBr15)	—	PCD probable
Br-44	3	28.7	Altered	Normal	na	—	PCD probable
Br-45	6*	11	1.7-Altered	Normal	nd	—	PCD probable
Br-46	8*	8.3	1.7-Static	Normal	na	—	PCD probable
Br-47	14, Dx	23.6	1.7-Red ampl	Normal	na	—	PCD probable
Br-48	9, Dx	84.3	1.7-Red ampl	Normal	na	—	Clinical PCD
Br-49	9, SI	41.9	Altered	Normal	na	—	Clinical PCD

Legend: *consanguineous; **the altered transmission electronic microscopy was used to determine the PDC diagnosis; ^π^Pac6 RPGR X linked mutations is rarely associated with respiratoy cilia defect; ^£^Pac7 CCDC39 is reduced or absent in CCDC40 loss of function mutation; ***Pac20: TEM: 20% of cilia with OIDA, the genetic variants were: *CFTR*^c,e^/*DNAH5*^c,e^/*DNAH11*^c,d^/*DYX1C1-CCPG1*^a,e^; nNO, nasal nitric oxide; nL/min, normal litres per minute; CBF: Cilia Beat Frequency; CBP: Cilia Beat Pattern; US, ultrastructure; TEM, transmission electronic microscopy; IF, immunofluorescence; SI, situs inversus; Dx, dextrocardia; CHD, cardiac heart disease; Ps, polysplenia; sn, sibling number; ODA, outer dynein arm; OIDA, outer inner dynein arm; MTD + IDA, microtubular disorganisation + inner dynein arm; APC, absence of central pair; ACP + T, absence of central pair + transposition; Red ampl, reduced amplitude; Inconcl, inconclusive; OBr: Omran-Brazil laboratory code; rs: repeat sample; na: not altered; nd: not done; *CCDC151*, Coiled-Coil Domain Containing 151; *CCDC39*, Coiled-Coil Domain Containing 39; *CCDC40*, Coiled-Coil Domain Containing 40; *DNAI2*, Dynein Axonemal Intermediate Chain 2; *DNAH5*, Dynein Axonemal Heavy Chain 5; *DNAH11*, Dynein Axonemal Heavy Chain 11; *DNAAF3*, Dynein Axonemal Assembly Factor 3; *DYX1C1-CCPG1*, Dyslexia Susceptibility 1 Candidate 1 and Cell Cycle Progression 1; *GAS8*, Growth Arrest Specific 8; *RSPH1*, Radial Spoke Head 1 Homolog; *RSPH9*, Radial Spoke Head 9 Homolog; *RPGR*, Retinitis Pigmentosa Gtpase Regulator X Cromossome, in hemizygous. Additional information about genetic screening: ^a^proved PCD variant; ^b^variant with probably pathogenic outcome on Polyphen predictor and/or deleterious outcome on Sift predictor; ^c^uncertain significance; ^d^homozygotes; ^e^heterozygotes; bold type, PCD diagnosis using genetic screening, also the other uncertain significance variants are shown in the Supplementary File (Table S5). The complete data regarding the genotypes divided by patient are presented in Supplementary File (Table S2) and Supplementary File (Table S5). Additionally, we included information about the genetic variants with proved pathogenicity in Supplementary File (Table S3) and data regarding the genetic variants with uncertain significance to pathogenicity in Supplementary File (Table S4). The patients Br-6 (X-linked gene), Br-20, Br-22, Br-39, Br-40 and Br-41 (homozygotes to uncertain significance mutations in PCD-causing genes) were considered to have a genetic PCD diagnosis, however, more studies should be performed to reach a better conclusion. Furthermore, the results may contain genetic variants of unknown significance, and a genetic diagnosis may not be clearly established. Thus, genetic counselling is recommended.

Data on nNO production rate measurements in control patients (127.3 ± 47.6 nL/min) and in non-PCD bronchiectatic patients (88.2 ± 27.4 nL/min) are shown in the Supplementary File (Table S1). In the entire PCD population, the mean ± SD nNO was 17.2 ± 15.6 nL/min (Table 3).

Thirty-five PCD patients and all controls underwent CBP evaluation. All the PCD patients presented altered exams, except for two (just one test: movement altered - Table 3).

**Figure 1:**
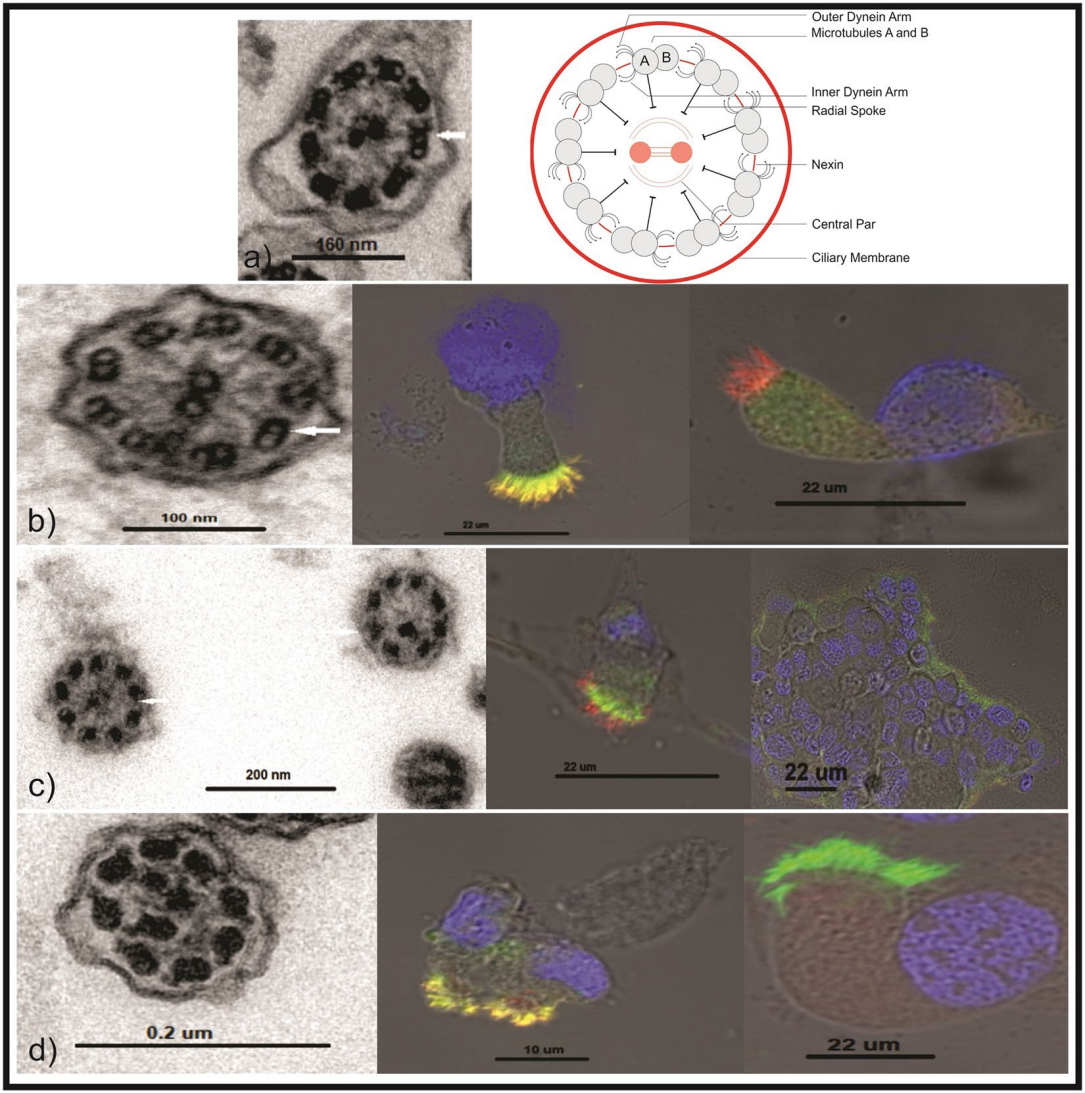
Examples of transmission electron microscopy (TEM) and immunofluorescence microscopy (IMF) results. (**a**) TEM cross-section of a control respiratory cilium, showing the typical “9 + 2” arrangement with nine outer-microtubule doublets and a central pair of microtubules (left panel) and a drawing of the respiratory motile cilium (right panel). (**b**) ODA arm defect shown by TEM (left panel) and IMF with antibodies directed against DNAH5 (green) and RSPH4A (red). In control cilia (middle panel), both proteins colocalize along the ciliary axonemes (yellow). Cilia with an ODA defect show absence of DNAH5 from the ciliary axonemes (right panel). (**c**) TEM section of a cilium with absent of central pair (9 + 0) and transposition defect (8 + 1) (left panel). IMF: antibodies directed against DNAH11 (green) and RSPH9 (red) (middle panel). Cilia with radial spoke defect show absence of RSPH9 (right panel). (**d**) Microtubular disorganisation + Inner Dynein Arm defect by TEM (left panel) and IMF microscopy using an antibody directed against CCDC39 (red) and DNAH5 (green). In the control cells (middle panel), CCDC39 (red) colocalizes with DNAH5 (green) along the ciliary axonemes (yellow). By contrast, cells from a PCD individual with tubular disorganisation CCDC39 was completely absent from the ciliary axonemes, indicating a CCDC39 defect (right panel). The nuclei were stained with Hoechst 3342 (blue).

Non-PCD bronchiectatic patients presented normal ultrastructure evaluations. Among the PCD cases, the brushing nose technique to collect cilia cells was initially successful in 46/49 cases (93.8%) with repetition of a second brush in three patients. In PCD patients, the results were as follows: dynein arm defects or dynein deficiency [DD: 20/49 (41%)], microtubular disorganisation + inner dynein arm (MTD + IDA) [14/49 (29%)], absence of central pair (ACP) plus or not transposition (ACP + T) [6/49 (12%)], and normal ultrastructure (NU) [7/49 (14%)] (Table 3, Fig. 1). Cilia IFM was performed on 28 patients, and the results were altered in 19/28 patients (68%) (Table 3, Fig. 1).

All PCD patients except two underwent genetic screening. Bi-allelic PCD pathogenic variants in autosomal recessive pattern inheritance were found in the following order: *CCDC40* [8/49 (16%) patients], *DNAH5* [4/49 (8%) patients], *RSPH1* and *DYX1C1* [3/49 (6%) patients], *DNAAF3* [2/49 (4.1%)], and 1/49 (2%) for the following genes: *CCDC39*, *DNAI2*, *DNAH11*, *RPGR* and *CCDC151*. An overview and considerations regarding the genetic screening in the use of pathogenic variants are shown in brief in Table 3, and the complete overview is shown in the Supplementary File (Table S2). The genetic variants with proven pathogenicity screened in patients with phenotypes compatible with PCD are described in the Supplementary File (Table S3), and the variants with uncertain significance are summarized in the Supplementary File (Table S4). Related genotypes screened in patients with phenotypes compatible with primary ciliary dyskinesia are described in the Supplementary File (Table S5).

**Figure 2:**
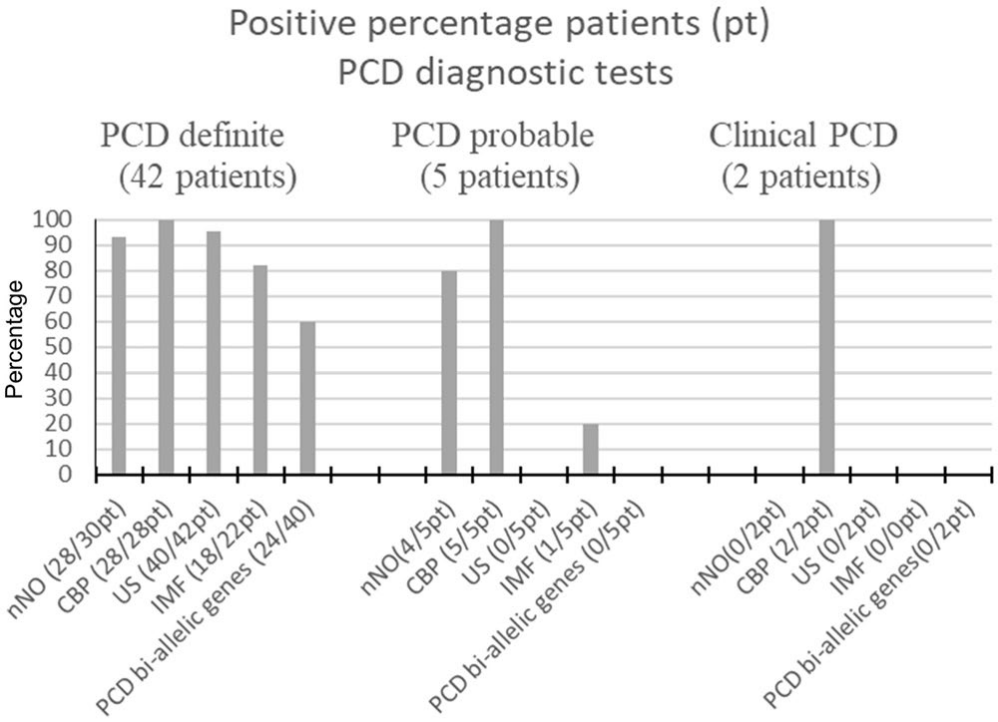
Results of positive PCD diagnostic tests.

According to the first results of the iPCD cohort^3^, this patient group was categorized as follows: definite PCD diagnosis in 42/49 (86%) patients, probable PCD in 5/49 (10%) patients, and clinical PCD in 2/49 (4%) patients. Table 3 shows the characteristics of each group. The nNO was 13.3 ± 9.4 nL/min in the definite PCD group; 22.2 ± 11.5 nL/min in the probable PCD group; and 63.1 ± 21.2 nL/min in the clinical PCD group. PCD diagnostic test results are summarized in Fig. 2.

### Assessment of severity

Twenty-five patients (51%) had severe lung impairment (FEV_1_ < 50%), with a FACED score of 3.4 ± 1.3 (range of one to six). At PCD diagnosis, eight patients had previously had lung transplants, four had been submitted to a lobectomy and one was oxygen-dependent. The more frequent ultrastructural abnormalities in these patients were as follows: DD in 11 patients, MTD + IDA in seven patients, ACP in three patients and NU in three patients.

### Inflammation and mucus rheology

EBC pH was collected from 35 patients (35/49, 71.4%), and the EBC pH of patients with PCD was 6.60 ± 0.33, which was less than that of a historical control group of healthy volunteers (7.7 ± 0.49)^14^. The mucus contact angle (53.2 ± 16.4°) was increased in relation to normal values (37 ± 2°) in 35/36 (97.2%) patients. Cough transportability (20.7 ± 5.8 mm) was below reference values (34 ± 9 mm) in 27/36 (75%) patients^15^. Viscosity values were 22.4 ± 7.7 cP, whereas plastic viscosity values were 10.9 ± 7.7 cP (no reference values available) (Table 4).

**Table 4:** Patients with primary ciliary dyskinesia who did not undergo lung transplantation and rheology studies.

Patient	PCD diagnosis	FACED	FEV_1_%	EBC pH	Contact angle	Cough	Plastic viscosity	Viscosity (cP)
Br-1	PCD	6	33	7.21	52.8	19.7	10.2	24.7
Br-2	PCD	5	45	6.09	56.4	29.7	20.9	12.1
Br-3	PCD	5	57	6.28	53.5	22.7	17.1	37.5
Br-4	PCD	1	64	6.82	47.8	25.7	8.8	19.9
Br-5	PCD	1	63	7.06	51.8	24.3	1.5	19.0
Br-6	PCD	1	62	6.40	50.1	14.3	0.0	16.5
Br-8	PCD	1	59	6.54	54.0	21.3	7.5	13.3
Br-9	PCD	4	32	6.76	47.8	19.0	7.0	17.5
Br-10	PCD	3	33	6.85	52.8	18.3	1.4	16.4
Br-11	PCD	3	19	6.60	48.1	20.7	28.4	12.6
Br-12	PCD	3	28	6.37	59.4	29.7	7.9	28.4
Br-13	PCD	3	29	7.03	51.7	14.7	12.1	25.5
Br-15	PCD	1	67	6.51	61.5	21.3	4.9	17.5
Br-16	PCD	2	70	6.47	55.8	25.3	4.1	17.5
Br-17	PCD	4	54	7.05	45.5	16.7	30.5	20.4
Br-18	PCD	2	47	6.59	47.4	21.0	28.0	15.7
Br-19	PCD	1	70	6.21	54.2	21.3	10.9	23.5
Br-20	PCD	2	42	6.00	52.0	14.3	24.1	34.1
Br-21*	PCD	4	68	6.48	59.6	33.0	9.0	24.1
Br-22	PCD	3	31	7.03	47.3	15.0	8.1	21.0
Br-23	PCD	3	56	6.30	142.9	15.7	8.1	40.2
Br-24*	PCD	5	18	6.42	54.7	14.7	11.8	40.4
Br-26	PCD	1	29	7.24	52.1	23.7	5.3	20.8
Br-27	PCD	3	36	6.35	53.4	17.7	9.8	22.2
Br-28	PCD	6	34	6.62	40.6	13.7	12.4	32.1
Br-29	PCD	1	55	6.47	47.1	27.5	4.7	14.0
Br-30	PCD	2	35	6.33	50.3	16.0	2.9	20.6
Br-31	PCD	2	63	7.11	50.0	8.5	8.0	28.5
Br-32	PCD	5	39	6.35	54.2	13.7	6.0	15.8
Br-33	PCD	2	64	6.79	44.0	27.0	7.0	28.3
Br-34	PCD	2	32	6.29	43.3	34.3	5.5	21.5
Br-40	PCD	2	59	6.70	31.4	17.7	12.9	14.6
Br-41	PCD	3	38	6.22	52.8	21.7	15.2	29.3
Br-44	Probable PCD	4	75	6.83	50.2	22.0	19.8	13.1
Br-47	Probable PCD	2	104	6.21	45.6	21.3	8.7	25.6

Legend: FEV_1_, Forced Expiratory Volume in one second; EBC, Exhaled breath condensate; *No lung transplantation at the time of evaluation.

## Discussion

In this study, we report the diagnostic assessment and the clinical, functional and genetic characteristics of an adult PCD population of 49 individuals in São Paulo, Brazil. Fifty-one percent of the patients presented severe functional impairment in adult life, corroborating the idea that PCD is not a mild disease^12^. This is the first characterization of a group of adult PCD patients in this country.

The newly diagnosed PCD patients in this study represented ten percent of the patients in the Bronchiectasis Outpatient Service of the largest tertiary care hospital in Brazil, which is in keeping with the frequency of other studies^16^, showing that PCD is not as rare as previously thought in Brazil. This population had a mean age of 41.6 ± 12.9 years, and 92% were Caucasians with relatively high rates (55%) of consanguinity, which remains frequent in this country. *Situs inversus* (50%) was the more frequent laterality disorder, in accordance with previous reports^17^.

The diagnosis of PCD remains challenging since none of the available tests can be used as a stand-alone test^18^. Referral centres differ in the combination of five tests used to assess diagnosis: nNO, HSVM, TEM, IFM and genetic tests^18^. We were able to perform all of these tests on 53% of the patients, and ultrastructure and/or genetic screening were performed on all patients. Few studies have evaluated the five tests in the same patient group^19,20^. Furthermore, differences between the North American^21^ and European diagnostic consensus^22^ increase the complexity of the diagnostic approach. In our setting, we consider the North American consensus more feasible since it requires fewer replicates of examinations. IFM contributed to diagnosis in 68% of the patients, suggesting that in medical settings where genetic screening is not affordable, this technique could represent a viable alternative. Some patients did not present positive IFM results because of inadequate samples due excess mucus or few cilia^6^. Therefore, we cannot exclude the possibility that the positivity of this technique could have been higher if we had repeated the exams.

Recent data indicate that PCD affects lung function in early life^23^. At the time of PCD diagnosis, 51% of our patients presented severe lung involvement. Eight (16%) patients had undergone lung transplantation at diagnosis, 4/49 (8%) had been lobectomized and 1/49 (2%) was oxygen dependent, confirming disease severity. In addition, a high prevalence of chronic cPa was present in this population, higher than previously reported^10,24^. Although dynein cilia defect was the most frequent abnormality found in the severe patients, the MTD + IDA defect was present in the younger (≤46 y) patients. These findings suggest that the MTD + IDA defect is associated with a more rapid decline in lung function^20^. There are few available data on the effect of early PCD diagnosis on later life lung function^23,25^. Nevertheless, it is highly likely that the lack of early PCD diagnosis and the lack of long-term, adequate and intensive treatment contributed to the disease severity in these patients.

Ultrastructure cilia defect indicated the diagnosis in 81.6% of the patients, and the genetic panel tests identified 49% (24/49) of the patients with the PCD bi-allelic gene. Therefore, genetic tests can only not be used to exclude the diagnosis of PCD^26^. Moreover, we found many variants of uncertain significance that could be associated with our genetically mixed population.

We observed phenotype-genotype inconsistencies in three patients (Table 3- Legend). So far, there has been no clear relationship between ultrastructure, genotypes, and respiratory phenotypes, mainly due to the clinical and genetic heterogeneity of PCD, and some inconsistencies are difficult to explain^27^. Interestingly, one male patient that had cilia ultrastructure compatible with MTD + IDA was hemizygous to a RPGR mutation linked to the X-chromosome. Such a mutation causes retinitis pigmentosa and is rarely associated with respiratory cilia defect^28,29^.

The PCD patients, like those with other chronic inflammatory airway diseases, presented lower EBC pH levels. It is possible that pH and other exhaled compounds could be a non-invasive tool to evaluate PCD treatment^30,31^. Our results indicate that reduced mucociliary transport, chronic inflammation and repeated infections in the respiratory tract produce a thicker mucus in PCD, as shown by the higher contact angle, reduced cough transportability and/or higher viscosity.

This study has several limitations. We acknowledge that our study population is small, and any conclusions should be drawn with care. However, this study represents an initial effort to adequately diagnose and fully characterize these patients in Brazil. It is possible that we have included more severe patients with bronchiectasis that were treated in a tertiary care centre. However, the prevalence of bronchiectasis in adult PCD patients seems to be very high^10^. The PICADAR scores were highly variable, with some patients presenting low scores, which could be explained by memory bias. We used an nNO handheld device for screening, which is less accurate^21^. However, in countries with limited resources, such as ours, the recommended chemiluminescence nNO analyser is generally not affordable. There is no standardization of equipment, samples, processing or analysis of cilia movement, and subtle abnormalities in CBP can be difficult to differentiate from secondary dyskinesia. In this study, CBP and nNO were used as accessory tools to strengthen the positive PCD diagnosis^18^.

In conclusion, we diagnosed and described the clinical condition of 49 adult PCD patients who were monitored at a Bronchiectasis service. This population was characterized by high consanguinity levels and severe pulmonary commitment. Genetically, a wide variability of pathogenic variants in genes related to PCD and variants of uncertain significance were found, which is likely to be a reflection of the genetically mixed population of Brazil. PCD is considered an orphan disease, as it has neither the prevalence of asthma nor the lethality of cystic fibrosis. We hope to use this series of patients to contribute to PCD awareness in our country and demonstrate the need for earlier diagnosis.

## Methods

This cross-sectional study included 55 adults with suspicion of PCD selected from 500 patients monitored at the Bronchiectasis Outpatient Service, Pulmonology Division, São Paulo University Medical School, from 2015 to 2017. This study was approved by the Ethics Committee of the institution [CAAE: 22823414.8.0000.0068]. All subjects signed written informed consent statements.

### Eligibility

Patients characterized with idiopathic bronchiectasis after a systematic aetiology protocol evaluation were screened for this study. Our institutional protocol includes genetic and/or sweat testing for cystic fibrosis, assessment of gastroesophageal reflux disorder, immunodeficiency (HIV and immunoglobulins) tests, alpha-1 anti-trypsin serum levels, rheumatological antibodies, white blood cell counts and sputum cultures (aerobic, fungi and mycobacteria). Patients were selected to enrol in the PCD diagnosis effort if they presented at least one of the following conditions: laterality disorders or productive chronic cough associated with recurring lower respiratory infections with or without upper respiratory infections and predominance of tomographic findings (bronchiectasis and tree in bud opacities) in the lower, middle and lingula lobes. Patients were submitted to at least two of the following diagnostic tests: nNO production rate measurement, cilia movement evaluation, ciliary ultrastructure evaluation, IFM and genetic tests.

We also evaluated a group of healthy volunteers for nNO measurements and cilia movement to validate our findings. Individuals were excluded from the control group if they had experienced respiratory symptoms in the previous month and/or had a history of smoking.

### Clinical characterization

We collected the following information: demographic variables including age, sex, self-reported race, place of birth, parental consanguinity, symptoms in relatives, body mass index (BMI), and pulmonary function tests. We obtained data on the presence of chronic *Pseudomonas aeruginosa colonization* (two or more isolates of the same organism at least three months apart in one year), spermiogram, and previous surgery interventions and/or oxygen dependence. Patients were further characterized by PICADAR^32^ and FACED scores^33^. In a subset of patients, exhaled breath condensate and mucus rheology were performed.

### Diagnostic assessment

#### Nasal nitric oxide production rate (nNO)

The nNO production rate measurements were made using a NIOX MINO (AEROCRINE AB^®^, Solna, Sweden) device, according to the manufacturer’s instructions^34^, in patients who had fasted for at least eight hours and were free from acute respiratory disease^34–36^. See the Supplementary File for methodological details. Control groups and patients with no PCD and details regarding the protocol were registered.

#### Cilia beat frequency and pattern (CBF and CBP) and ciliary ultrastructure

Cilia were collected to study movement and ultrastructure procedures. The detailed method is described in the Supplementary File^13^.

#### Evaluation of CBF and CBP

Several strips of ciliated epithelium movement were recorded for each patient. The recorded cilia cell movement videos were studied a second time, and the CBF and CBP were classified as follows: recognisability of regular forward and recovery strokes (normal), static cilia, almost static cilia with minimal residual movement, stiff beating due to a reduced bending capacity/amplitude, and abnormal circular beating^37,38^. Cilia movement with no agreement with any previous description but without an effective stroke were considered altered. Only strips of ciliated epithelium without damaged epithelium and no isolated cilia cells were evaluated. CBF was evaluated according to previous studies^13^, and the final results of 10 measurements were recorded. In our study, we assumed CBP to be more important than CBF for evaluating and defining movement^39^. Therefore, if the CBP was altered, we assumed the final result of the movement to be altered.

#### Analyses of ciliary Ultrastructure by TEM

The collected material was immersed in 2% glutaraldehyde and processed according to standardized norms^40^ using cross-section thicknesses of 50 nm. Quantitative and qualitative analyses were conducted. At least 100 cross-sections of cilia were evaluated. High-quality cross-sections were assessed for the presence of dynein arms. Details of the TEM evaluation are described in the Supplementary File^41–43^.

#### High-Resolution Immunofluorescence Microscopy (IFM)

High-resolution IFM was performed at the University Hospital of Muenster, Germany, following the laboratory protocols. The following antibodies were used: anti-DNAH5, anti-RSPH4A, anti-RSPH9, anti-CCDC39, anti-GAS8 and anti-DNAH11^6^. High-resolution immunofluorescence images were taken using a Zeiss Apotome Axiovert 200 (processed with AxioVision 4.8) or Zeiss LSM880 (processed with ZEN2 software) (see the Supplementary File).

#### Genetics analysis

Detailed methodologies used for the genetic analysis are described in the Supplementary File. DNA extraction was performed using the FlexiGene DNA Kit extraction kit (Qiagen^®^, Valencia, CA, 91355, USA). After DNA extraction, the sample was quantified in Qubit 2.0 (Life Technologies^®^, São Paulo/SP, Brazil) and then submitted to panel sequencing.

#### Pulmonary function test (PFT)

Spirometry (Koko Legend, Inspire Health Inc., Longmont, USA) was performed according to the recommendations of the American Thoracic Society and the European Respiratory Society. Data were interpreted based on the methods proposed by Pereira and collaborators who examined the Brazilian population^44^. The forced vital capacity (FVC) and FEV_1_ were considered PFT parameters.

#### PCD diagnosis and severity assessment

The possibility of having PCD was considered for patients who had altered results in at least two of the following tests: nNO production rate measurement, evaluation of ciliary movement (CBP), ultrastructure analyses of the cilia (TEM), IFM and genetic tests related to PCD gene sequencing. Patients with confirmed ciliary ultrastructural defect and/or with bi-allelic causing-PCD gene variants were definitively diagnosed with PCD^3,4,39^. Probable PCD was assumed for patients with only one abnormal test, such as altered movement (CBP) and/or low nNO production rate compared to the control the group, but all typical clinical symptoms were present. Patients with negative or ambiguous tests but strong clinical characteristics were defined as a clinical PCD diagnosis^3^.

We considered patients to have severe disease when the forced expiratory volume in one second (FEV_1_) was < 50% of that predicted.

#### pH in exhaled breath condensate (EBC)

The patients fasted for at least eight hours before EBC collection. For EBC collection and analysis, see the Supplementary File. Immediately after EBC collection, the EBC pH was analysed, and the results were compared with historical normal values (7.7 ± 0.49)^14^.

#### Mucus rheology

Patients were asked to cough three times, and the sputum samples were stored at −80 °C for further analyses of contact angle, cough transportability and (plastic) viscosity. For the methodological details, see the Supplementary File^15^.

### Statements

#### Approval

Written approval was obtained from the patients for re search purposes, and from the institutional ethical committee (Comissão de Ética para Análise de Projetos de Pesquisa – CAPPesq).

#### Accordance

The methods were used in accordance with the relevant guidelines and regulations.

#### Informed Consent

An informed consent was obtained from all participants.

## Supplementary information

Supplementary Information Materials

## Data Availability

All the data generated or analysed during this study are included in this published article, and its Supplementary Information Files.
