# Supplementary material for: Severe pulmonary disease in an adult primary ciliary dyskinesia population in Brazil
Source: Sci Rep. 2019 Jun 18;9:8693. doi: 10.1038/s41598-019-45017-1 (PMC6582273; doi:10.1038/s41598-019-45017-1)
Supplement: Supplementary file 1 — Supplementary Information Materials [file 41598_2019_45017_MOESM1_ESM.docx]

Supplementary Information Materials

Severe pulmonary disease in an adult primary ciliary dyskinesia population in Brazil

Mary Anne Kowal Olm^1,*,+^; Fernando Augusto Lima Marson^2,+^; Rodrigo Abensur Athanazio^3^; Naomi Kondo Nakagawa^1^; Mariangela Macchione^1^; Niki Tomas Loges^4^; Heymut Omran^4^; Samia Zahi Rached^3^; Carmen Sílvia Bertuzzo^2^; Rafael Stelmach^3^; Paulo Hilário Nascimento Saldiva^1^; José Dirceu Ribeiro^2,5^; Marcus Herbert Jones^6^; Thais Mauad^1^

+, equally contributing authors

1. Department of Pathology, São Paulo University Medical School, São Paulo, SP, 01246-903, Brazil.

2. Department of Medical Genetics and Genomic Medicine, Faculty of Medical Science, University of Campinas, Campinas, SP, 13083-887, Brazil

3. Pulmonary Division, Heart Institute (InCor), Hospital das Clínicas da Faculdade de Medicina da Universidade de São Paulo, SP, 05403-000, Brazil

4. Department of Pediatrics and General Pediatrics, Muenster University Hospital, Muenster, 48149, Germany

5. Department of Pediatrics, Faculty of Medical Science, University of Campinas, Campinas, SP, 13083-887, Brazil

6. Department of Pediatrics, Pontifical Catholic University of Rio Grande do Sul, Porto Alegre, RS, 90610-000, Brazil

Correspondence to:

Mary Anne Kowal Olm

Av Dr Arnaldo 455 room 1155

Department of Pathology

School of Medicine, São Paulo University

CEP 01246-903- São Paulo- SP, Brazil

e-mail: maryakolm@yahoo.com.br

METHODS

Nasal nitric oxide production rate (nNO)

Analyses were performed between seven and nine a.m. The room temperature and humidity were in accordance with the manufacturer’s recommendations. The procedure for measuring the nNO production rate consisted of relaxed tidal breathing through the mouth during sampling, with a flow rate of five mL/s for adults (1). Two 45-sec measurements were taken from the same nostril of each patient, and the mean ppb (parts per billion) was converted to nL/min according to the flow rate used (ppbX0.3L). The handheld device was chosen based on previous studies (2) and considering local difficulties to acquire the stationary device; palate resistance technic cut-off values of <30 nL/min were considered compatible with PCD diagnosis (1).

Ciliary beat pattern (CBP) and ultrastructure

Cilia collection for studying movement and ultrastructure procedures

Disposable brushes were used to collect cilia cells for the cilia movement study (Edel White S^®^ 0.6 mm/3 mm Swiss) and for the TEM procedure (BC-202D-3010 Olympus^®^ Japan). The brushes were lightly lubricated with distilled water (at room temperature) beforehand, and a circular movement with the brushes was performed through the inferior turbinate. The collected cilia cells were spread over glass slides, immersed in Ringer’s solution at 37°C and covered with thin slides. The temperature of the slides was kept under control (a plate with a digital temperature control was attached to the microscope Axio Imager^®^). The videos were recorded within 20 min and at 100 frames per second (3), and videos of movement were produced. The patients and control group answered negatively when questioned if they had signs of acute respiratory distress in the last month.

Ultrastructural analyses by TEM

Formvar grids were not used to enhance grid visualization. Each square of the grids was evaluated methodically. Outer dynein arm defects were understood as either the absence of outer dynein arms or the presence of short, stub-like projections rather than fully developed arms. Microtubular disorganisation plus inner dynein arm was understood as total disorganisation of microtubules, coupled with an absence of inner dynein arms. Transposition was understood as an absence of the central pair in some transverse cross-sections, which was replaced by an outer microtubular doublet in other cross-sections(4). The number of outer and inner arms was counted on cilia with few dynein arms, and the mean was calculated. Altered ultrastructure was considered in the following cases: absence or shortening of outer/inner dynein arm; disorganisation of microtubes with or without the absence of the inner dynein arm; and absence of the central pair with or without transposition. Mean counts lower than 1.6 for outer dynein arms and lower than 0.6 for inner dynein arms were considered to indicate PCD (5,6) if the [nNO], IFM or genetic test were also altered.

High-resolution immunofluorescence microscopy (IFM)

Respiratory epithelial cells were obtained by nasal brushing and suspended in cell culture

medium (RPMI Medium 1640, Glutamine, HEPES, Life Technologies^®^). Samples were spread onto glass slides, air-dried, and sent to the University Hospital Muenster. The following protocol was used to prepare the slides for high-resolution IFM: the slides were first defrosted, and the cells were washed with phosphate buffered saline (PBS) solution for five min. The cells were then incubated with 4% PFA-Paraformaldehyde (pH 7) for 15 min, and the slides were washed three times with 1x PBS. Next, the cells were incubated in 0.2% TritonX-100 for 15 min and then washed three times with PBS solution. The cells were incubated overnight at 4°C in 1% blocking solution (skim milk). The blocking solution was used to dilute the primary and secondary antibodies.

The slides were washed the next morning with 1xPBS solution, and the cells were incubated with primary antibody dilution overnight at 4°C. After incubation, the antibody solution was removed, and the slides were washed five times with 1x PBS.

The cells were incubated for 30 min at room temperature with secondary antibody dilution (#2041: 1:1,000) and then washed five times with 1x PBS solution. Then, the cells were incubated for 10 min at room temperature with Hoechst33342 dye (200p1; 1:1,000 in PBS solution) and washed five times with 1xPBS solution.

The 1x PBS was removed, and the slides were dried. Two drops of Dako Faramount Mounting Medium were added, and the cells were covered with a slide and pressed to remove air bubbles. The slides were then allowed to dry slowly overnight. The following antibodies were used: anti-DNAH5 (Dynein Axonemal Heavy Chain 5), anti RSPH4A (Radial Spoke Head 4 Protein 4 Homolog), anti-RSPH9 (Radial Spoke Head Protein 9 Homolog), anti-RSPH4A (Radial Spoke Head Protein 4 Homolog), anti-CCDC39 (Coiled-Coil Domain Containing 39), anti-GAS8 (Growth Arrest Specific 8) and anti-DNAH11 (Dynein Axonemal Heavy Chain 11).

Genetic Tests

Blood collection and DNA extraction

Four millilitres of blood were collected in a tube containing EDTA (Ethylenediamine tetra-acetic acid). Two tubes of blood were collected for each patient. After collection, the material was kept at room temperature (25°C) and transported to the laboratory of Molecular Genetics, Campinas, São Paulo, University of Campinas. In the laboratory, the blood sample was recorded for individual coding, and the material was processed for DNA extraction. DNA extraction was performed using the FlexiGene DNA extraction kit (Qiagen^®^, Valencia, CA, 91355, USA). After DNA extraction, the sample was quantified in Qubit 2.0 (Life Technologies^®^, São Paulo/SP, Brazil), and then a sample was sent to panel sequencing.

DNA library preparation

The DNA libraries of the patients were built, along with the positive and negative controls, as per the protocol (#1000000002191v04) made available by the supplier (Illumina^®^, San Diego, CA‎, USA – all described reagents were obtained from the company through standard protocol) including the following phases:

*(i) preparation of the input:* the DNA samples were quantified by fluorimetry in Qubit^®^ 2.0 (Life Technologies^®^, Carlsbad, CA, USA) with the use of the Qubit^®^ kit dsDNA HS (high sensitivity) assay (Q32851, Life Technologies^®^) and diluted in H_2_O to a concentration of 10 ng/µL. The samples were quantified again for preparation of the input of 10 ng in four µL of resuspension solution 1 (2.5 ng/µL). After the procedure, one µL of sample stabilization solution 1 was added.

*(ii) hybridization of oligonucleotide:* hybridization of the oligonucleotides pool (upstream and downstream) specific to the regions of interest in a Veriti 96-Well Thermal Cycler (Applied Biosystems^®^, Waltham, MA, USA).

*(iii) removal of the* *oligonucleotides in suspension:* the separation and removal of the oligonucleotides in suspension from the other components of the solution were performed with the use of magnetic beads with affinity to genomic DNA.

*(iv) oligonucleotide extension and ligation:* DNA polymerase replicates the DNA sequence from the upstream oligonucleotide (3' end) towards the region of interest, limited by the downstream oligonucleotide (5' end) by DNA ligase. The reaction product contains the regions of interest flanked by the sequences necessary for amplification.

*(v) amplification of DNA libraries:* amplification was performed in 29 cycles, according to the number of amplicons, as per the manufacturer's protocol.

*(vi) DNA library clean-up:* similar to step (iii), with the use of magnetic beads to separate polymerase chain reaction products from other components in the reaction solution. After removal, the product was evaluated in 4% agarose gel and submitted to electrophoresis to identify the amplified fragments.

*(vii) DNA library normalization:* the libraries were normalized to a similar concentration amongst themselves to reduce the likelihood of non-homogeneous sequencing amongst the products. This process occurred through beads that bind to DNA to their saturation point.

*(viii) denaturation and dilution of DNA libraries***:** 15 µL of the DNA library pool was diluted in 585 µL of hybridization buffer 1, as per the technical support from Illumina. The DNA library pool was denatured by incubation for two min on a plate preheated to 98°C, followed by five min at zero degrees – with the subsequent application on the flow cell.

Cluster generation and sequencing of DNA libraries

For cluster generation and sequencing of DNA libraries from the patients, we used the MiSeq^®^ equipment and a MiSeq Reagent Kit^®^ v2 and PE MiSeq Flow Cell^®^.

*(i) cluster generation:* DNA molecules, in a sample tube, bind to the flow cell surface through complementarity to adapters that are fixed at the ends. Thus, amplification occurs in these areas through the formation of bridges, until the flow cell is full of copies of the region of interest.

*(ii) sequencing of DNA libraries:* sequencing was performed using a TruSeq^®^ 202 amplicon custom panel. Data were tabulated through a sample worksheet customization – sample sheet and the amplicon-identifying file – manifest file. The following genes were analysed in our study: Armadillo Repeat Containing (*ARMC4*); Chromosome 21 Open Reading Frame 59 (*C21ORF59*); Coiled-Coil Domain Containing 103 (*CCDC103*); Coiled-Coil Domain Containing 114 (*CCDC114*); Coiled-Coil Domain Containing 151 (*CCDC151*); *CCDC39*; Coiled-Coil Domain Containing 40 (*CCDC40*); Coiled-Coil Domain Containing 65 (*CCDC65*); Cyclin O (*CCNO*); Dynein, Axonemal, Assembly Factor 1 (*DNAAF1*); Dynein, Axonemal, Assembly Factor 2 (*DNAAF2*); Dynein, Axonemal, Assembly Factor 3 (*DNAAF3*); *DNAH11*; *DNAH5*; Dynein, Axonemal, Intermediate Chain 1 (*DNAI1*); Dynein, Axonemal, Intermediate Chain 2 (*DNAI2*); Dynein, Axonemal, Light Chain 1 (*DNAL1*); Dynein Regulatory Complex Subunit 1 (*DRC1*); Dyslexia Susceptibility 1 Candidate 1 (*DYX1C1*); HEAT Repeat Containing 2 (*HEATR2*); Axonemal Central Pair Apparatus Protein (*HYDIN*); Leucine Rich Repeat Containing 6 (*LRRC6*); NME/NM23 Family Member 8 (*NME8*); Oral-Facial-Digital Syndrome 1 (*OFD1*); Retinitis Pigmentosa Gtpase Regulator (*RPGR*); Radial Spoke Head 1 Homolog (*Chlamydomonas*) (*RSPH1*); Radial Spoke Head 4 Homolog A (*Chlamydomonas*) (*RSPH4A*); *RSPH9*; Sperm Associated Antigen 1 (*SPAG1*); Zinc Finger, MYND-Type Containing 10 (*ZMYND10*); and Coiled-Coil Domain Containing 164 (*CCDC164*). In addition, we performed genetic sequencing of the *CFTR* (Cystic Fibrosis Transmembrane Conductance Regulator) gene to exclude cystic fibrosis disease.

Acquisition of data from the sequencing of DNA libraries

The results were obtained in the BaseSpace Sequence Hub^®^ (Illumina^®^), which is a cloud computing tool developed for the collection and analysis of sequencing data. In addition to its applications, BaseSpace hosts commercial versions from other developers, which promotes versatility.

Alignment was performed using the TruSeq Amplicon^®^ version 3.0 (Illumina^®^) – available in a virtual environment – with the use of the Smith-Waterman (1981) (7) algorithm in regions delimited by the custom manifest file. Variant calls and annotations were performed in the Illumina Variant Studio^®^ v3.0 (Illumina^®^) (.vcf, Variant Call Format). Variants identified as likely pathogenic were confirmed visually in the Integrative Genomics Viewer^®^ (IGV) version 2.4 (Broad Institute, Cambridge, MA, USA), having the Human Genome 19 (hg19) as the base genome ^8–10^.

Classification of genetic variants

The classification of variants as pathogenic considered some of the consensus criteria of the “American College of Medical Genetics and Genomics” and the “Association for Molecular Pathology” (10).

The variants, when described in the specific databases, supported by functional analyses of valid biological significance, had greater evidence of pathogenicity. In general, when related to Mendelian disorders, allele frequency is considered to be a strong indication for a benign interpretation, as well as when the variants are identified in adult and healthy individuals. Additionally, for variants of uncertain significance, the following databases were consulted: (a) ClinVar [(https://www.ncbi.nlm.nih.gov/clinvar/)](https://www.ncbi.nlm.nih.gov/clinvar/), which is a free access database containing information about the interaction genetic variants with clinical phenotypes that have significance at the clinical, research or exclusively literary level; (b) InterVar (<http://wintervar.wglab.org/>), which is a bioinformatics tool for the clinical interpretation of genetic variants that considers the consensus of the “American College of Medical Genetics and Genomics” and the “Association for Molecular Pathology” ^12,13^, using the following classification: (i) benign; (ii) likely benign; (iii) uncertain significance; (iv) likely pathogenic; and (v) pathogenic.

pH in Exhaled breath condensate (EBC)

Samples were deaerated with 99.9% ultrapure argon gas (Gama Gases Ltda., São Paulo, Brazil) at a flow rate of 350 mL/min for 15 min to remove carbon dioxide. A pH meter (827 pH Lab, Metrohm Ltd., Herisau, Switzerland) was calibrated, and the pH was measured with a microelectrode. Three pH measurements were taken, and the final result was the mean of the 3 values. The EBC pH results were compared with normal values (7.7±0.49) (12). EBC pH correlated well with other indices of airway inflammatory in acute exacerbations of asthma, cystic fibrosis, and chronic obstructive pulmonary disease.

After rising the mouth with distilled water, the patients breathed through an acrylic collecting device surrounded by dry ice (-75°C) for 15 min with a normal tidal volume (350 to 500 mL) and respiratory rate for approximately 12 to 16 min, which resulted in one to 1.5 mL of EBC fluid.

Mucus rheology

The contact angle measures the wettability, which is the biological capacity of a fluid to spread onto a flat surface, as previously described (12). Sputum samples were placed on a glass slide surface that was previously treated with sulphocromic acid; measurements were performed with the aid of an optical microscope (25X) with a goniometer (scale 0 to 180°). The normal values of the contact angle were described between (37±2°) (12).

The sputum transportability by cough was analysed using a simulated cough machine that mimicked the cough reflex. The system consisted of a cylinder of compressed air connected to a cylindrical acrylic tube (four mm in internal diameter and 133 mm in length) that released compressed air with an airflow of 235 L/min (13). Each sputum sample (50 µL) was processed only once, and the results of three samples were determined in millimetres (mm). The normal mean values described for cough transportability are 34 ± 9 mm (13).

Viscosity and plastic viscosity

Viscosity is defined as the resistance of a fluid to flow and is measured as the ratio of the shear stress to shear rate in specific conditions. We performed viscosity analysis using a multi-speed viscometer (CAP 2000 model, Brookfield, Inc., USA). The absolute viscosity and plastic viscosity of 250 µL of the sputum sample were analysed under controlled laboratorial ambient conditions (25°C and 60% relative humidity) for three min with a speed range between 100 and 1000 r.p.m., timed for 30 sec in each speed. Plastic viscosity was measured (centipoise-cP) by calculating the difference between the dial readings of sputum at the two highest speeds.

Statistical analysis

In our study, the exploratory statistical tests were not considered important in a descriptive article like ours.

RESULTS

Control and non-PCD patient characterization

Table S1- Control group and excluded PCD subjects by the absence of clinical and laboratory information.

| Patient (P) and  control (C) | Age (y) | Sex | Nasal nitric  oxide (nL/min) | CBF (Hz) -  CBP | Ultrastructure  (TEM) |
| --- | --- | --- | --- | --- | --- |
| C-1 | 60 | F | 179.9 | (15.0) - Effective | - |
| C-2 | 50 | M | 130.8 | (9.2) - Effective | - |
| C-3 | 47 | M | 152.1 | (5.0) - Effective | - |
| C-4 | 53 | F | 67.1 | (9.2) - Effective | - |
| C-5 | 56 | F | 212.1 | (9.2) - Effective | - |
| C-6 | 60 | M | 103.4 | (11.8) - Effective | - |
| C-7 | 35 | F | 89.4 | (10.6) - Effective | - |
| C-8 | 34 | M | 83.9 | 10.0 - Effective | - |
| PCD | 56 | F | 64.5 | (6.7) - Effective | Normal |
| PCD | 53 | F | 57.7 | (8.3) - SCD | Normal |
| PCD | 27 | F | 95.9 | ( - ) - SCD | Normal |
| PCD | 47 | F | 107.6 | (8.3) - Effective | Normal |
| PCD | 49 | F | 68.3 | (13.3) - Effective | Normal |
| PCD | 28 | M | 135 | (10.6) - Effective | Normal |

Legend: y, years; M, male; F, female; nL/min, normal litres per minute; TEM, transmission electronic microscopy; SDC, secondary dyskinesia considered; CBF, cilia beat frequency; CBP, cilia beat pattern. (Control Group): nNO = 127.3 ± 47.6 nL/min; (Non-PCD Group): nNO = 88.2 ± 27.4 nL/min; CBF control group: 9.6 ± 2.6 Hz; (CBF Non-PCD patients): 8.3 ± 2.3 Hz.SDC: SecondaryClia Dyskinesia Data shown as the mean ± SD.

Table S2. Results from the tools used to determine the diagnosis of patients with phenotypes compatible with primary ciliary dyskinesia (PCD).

| Patient | Gene | Diagnosis |
| --- | --- | --- |
| Br-1 | *DNAI1*^b,e^/*HYDIN* ^c,e^ | PCD |
| Br-2** | *RSPH1*^a,f^ | PCD |
| Br-3 | *DNAI2*^a,d^ | PCD |
| Br-4 | *CFTR*^a,e^/*DNAH11*^c,f^/ *DYX1C1-CCPG1*^c,f^ | PCD |
| Br-5 | *CFTR*^b,e^/*DNAH5*^c,f^ | PCD |
| Br-6 | *CCDC39*^c,e^/*RPGR*(X-chromosome)^c,g^ | PCD |
| Br-7 | *CCDC40*^a,d^ | PCD |
| Br-8 | *CCDC40*^a,d^/*DNAH11*^a,e^ | PCD |
| Br-9 | *CCDC40*^c,e^/*HYDIN*^c,f^ | PCD |
| Br-10 | *CCDC39*^a,d^ | PCD |
| Br-11 | *CCDC40*^a,d^ | PCD |
| Br-12 | *CCDC151*^a,d^ | PCD |
| Br-13 | *DNAH5*^b,e^ | PCD |
| Br-14 | *DNAAF3*^a,d^ | PCD |
| Br-15 | *DNAH11*^c,e^/*DYX1C1-CCPG1^a^*^,e^ | PCD |
| Br-16 | *CCDC40*^a,d^ | PCD |
| Br-17 | *RSPH1*^a,d^ | PCD |
| Br-18 | *DNAH5* ^a,f^ | PCD |
| Br-19 | *DNAH5*^c,e^/*DYX1C1-CCPG1*^a,e^ | PCD |
| Br-20 | *CFTR*^c,e^/*DNAH5*^c,e^/*DNAH11*^c,d^/*DYX1C1-CCPG1*^a,e^ | PCD |
| Br-21 | *DNAH5*^a,d^ | PCD |
| Br-22 | *DNAAF*3^c,d^/*CCDC39*^c,e^ | PCD |
| Br-23 | *RSPH1*^a,d^ | PCD |
| Br-24 | *DNAH5*^a,d^ | PCD |
| Br-25 | *CCDC40*^a,d^ | PCD |
| Br-26 | *DNAH5*^a,d^ | PCD |
| Br-27 | *DNAH5*^a,d^ | PCD |
| Br-28 | *CCDC40*^a,d^ | PCD |
| Br-29 | *RSPH1*^a,d^ | PCD |
| Br-30** | *CFTR*^b,e^/*DNAH5*^c,f^ | PCD |
| Br-31** | *DNAH5*^c,f^ | PCD |
| Br-32 | *CCDC40*^a,d^ | PCD |
| Br-33 | *DNAH5*^a,f^ | PCD |
| Br-34** | *DNAH5*^b,e^ | PCD |
| Br-35 | *CCDC40*^a,d^ | PCD |
| Br-36 | Not consent | PCD |
| Br-37 | *RSPH1*^a,f^ | PCD |
| Br-38 | Not consent | PCD |
| Br-39 | *DYX1C1-CCPG1*^c,d^/*HDYN*^c,e^ | PCD |
| Br-40 | *CCDC40*^b,e^*/DYX1C1-CCGP1*^b,d^/*DNAH11*^c,e^/*DNAL1*^c,e^ | PCD |
| Br-41 | *DYX1C1-CCGP1*^b,d^/*DNAH11*^c,e^/*DNAL1*^c,e^ | PCD |
| Br-42** | *CFTR*^b,e^/ *DNAAF1*^c,e^/*DNAAF3*^c,e^/*DNAH1*^c,e^/*DNAH8*^c,e^/*HYDIN*^c,e^ | PCD |
| Br-43 | *DNAH1*^c,f^/*DNAH5*^c,e^ | PCD probable |
| Br-44 | *DNAH1*^c,e^/*HYDIN*^a,e+c,e^ | PCD probable |
| Br-45 | *RSPH1*^a,e^/*HYDIN*^c,e^ | PCD probable |
| Br-46 | *DNAH11*^c,e^/*HYDIN*^a,e^ | PCD probable |
| Br-47 | Absence of variants | PCD probable |
| Br-48 | *DNAH1*^c,f^/*DNAH11*^c,f^/*HYDIN*^c,e^ | Clinical PCD |
| Br-49 | *ARMC4*^a,e+c,e^/*DNAH1*^c,e^/*DRC1*^b,e^ | Clinical PCD |

Legend: **, the altered transmission electronic microscopy was used to determine the PDC diagnosis; *ARMC4*, Armadillo Repeat Containing; *CCDC151*, Coiled-Coil Domain Containing 151; *CCDC39*, Coiled-Coil Domain Containing 39; *CCDC40*, Coiled-Coil Domain Containing 40; *CFTR*, Cystic Fibrosis Transmembrane Conductance Regulator; *HYDIN*, Axonemal Central Pair Apparatus Protein; *DNAI2*, Dynein Axonemal Intermediate Chain 2; *DNAH5*, Dynein Axonemal Heavy Chain 5; *DNAH8*, Dynein Axonemal Heavy Chain 8; *DNAH11*, Dynein Axonemal Heavy Chain 11; *DNAI1*, Dynein, Axonemal, Intermediate Chain 1; *DNAL1*, Dynein, Axonemal, Light Chain 1; *DRC1*, Dynein Regulatory Complex Subunit 1; *DNAAF3*, Dynein Axonemal Assembly Factor 3; *DYX1C1-CCPG1*, Dyslexia Susceptibility 1 Candidate 1 and Cell Cycle Progression 1; *GAS8*, Growth Arrest Specific 8; *RSPH1*, Radial Spoke Head 1 Homolog; *RSPH9*, Radial Spoke Head 9 Homolog; *RPGR*, Retinitis Pigmentosa Gtpase Regulator. Additional information about genetic screening: ^a^, proved PCD variant; ^b^, variant with probably pathogenic outcome on Polyphen predictor and/or deleterious outcome on Sift predictor; ^c^, uncertain significance; **^d^,** homozygotes; ^e^, heterozygotes; ^f^, compound heterozygotes; ^g^, hemizygous; bold type, PCD diagnosis using genetic screening, also the other uncertain significance variants are shown in Supplementary File (Table S2) and Supplementary File (Table S5). Additionally, we included information about the genetic variants with proved pathogenicity in Supplementary File (Table S3) and data regarding the genetic variants with uncertain significance to pathogenicity in Supplementary File (Table S4). The patients Br-6 (X-linked gene), Br-20, Br-22, Br-39, Br-40 and Br-41 (homozygotes to uncertain significance mutations in PCD-causing genes) were considered to have a genetic PCD diagnosis, however, more studies should be performed to reach a better conclusion. Furthermore, the results may contain genetic variants of unknown significance, and a genetic diagnosis may not be clearly established. Thus, genetic counselling is recommended.

Table S3**.** Complete information regarding the genetic variants with proven pathogenicity screened in patients with phenotypes compatible with primary ciliary dyskinesia.

| Gene | N | Chr | HGVSc | HGVSp | dbSNP ID | Type | Consequence |
| --- | --- | --- | --- | --- | --- | --- | --- |
| *ARMC4* | 1 | 10 | NM_018076.2:c.97G>T | NP_060546.2:p.Glu33Ter | rs752348839 | SNV | Stop gained |
| *CCDC151* | 2 | 19 | NM_145045.4:c.583_595dupGCGCAAAACAGAC | NP_659482.3:p.His199ArgfsTer60 | rs750658321 | Insertion | Frameshift |
| *CCDC39* | 2 | 3 | NM_181426.1:c.2497_2498delCA | NP_852091.1:p.Gln833ValfsTer6 | - | Deletion | Frameshift, feature truncation |
| *CCDC40* | 2 | 17 | NM_017950.3:c.1232dupT | NP_060420.2:p.Met411IlefsTer3 | - | Insertion | Frameshift |
|  | 6 | 17 | NM_017950.3:c.1571delA | NP_060420.2:p.Gln524ArgfsTer19 | rs761619279 | Deletion | Frameshift |
|  | 4 | 17 | NM_017950.3:c.1989+1G>A | - | rs745993158 | SNV | Splice donor |
|  | 2 | 17 | NM_017950.3:c.2920C>T | NP_060420.2:p.Gln974Ter | - | SNV | Stop gained |
|  | 2 | 17 | NM_017950.3:c.3097A>T | NP_060420.2:p.Lys1033Ter | rs863224519 | SNV | Stop gained |
| *CFTR^a^* | 1 | 7 | NM_000492.3:c.350G>A | NP_000483.3:p.Arg117His | rs78655421 | SNV | Missense |
| *DNAAF3* | 2 | 19 | NM_001256714.1:c.1201dupG | NP_001243643.1:p.Asp401GlyfsTer63 | rs756430359 | Insertion | Frameshift |
| *DNAH11^b^* | 1 | 7 | NM_001277115.1:c.8533C>G | NP_001264044.1:p.Arg2845Gly | rs121908854 | SNV | Missense |
| *DNAH5* | 4 | 5 | NM_001369.2:c.13060delG | NP_001360.1:p.Ala4354ArgfsTer23 | - | Deletion | Frameshift |
|  | 1 | 5 | NM_001369.2:c.13458dupT | NP_001360.1:p.Asn4487Ter | rs775696136 | Insertion | Frameshift |
|  | 1 | 5 | NM_001369.2:c.11653C>T | NP_001360.1:p.Arg3885Ter | rs756032160 | SNV | Stop gained |
|  | 3 | 5 | NM_001369.2:c.13486C>T | NP_001360.1:p.Arg4496Ter | rs200901816 | SNV | Stop gained |
|  | 1 | 5 | NM_001369.2:c.2090T>A | NP_001360.1:p.Leu697Ter | - | SNV | Stop gained |
|  | 2 | 5 | NM_001369.2:c.4237C>T | NP_001360.1:p.Gln1413Ter | rs752734772 | SNV | Stop gained |
| *DNAI2* | 2 | 17 | NM_023036.4:c.787C>T | NP_075462.3:p.Arg263Ter | rs137852998 | SNV | Stop gained |
| *DYX1C1-CCPG1* | 3 | 15 | NM_130810.3:c.862_866delAAGAA | NP_570722.2:p.Lys288ProfsTer7 | - | Deletion | Frameshift |
| *HYDIN* | 1 | 16 | NM_001270974.1:c.11617dupA | NP_001257903.1:p.Ser3873LysfsTer81 | - | Insertion | Frameshift |
| *HYDIN* | 1 | 16 | NM_001270974.1:c.8675delA | NP_001257903.1:p.Gln2892ArgfsTer3 | - | Deletion | Frameshift, splice region |
| *RSPH1* | 2 | 21 | NM_080860.2:c.275-2A>C | - | rs151107532 | SNV | Splice acceptor |
|  | 1 | 21 | NM_080860.2:c.281G>A | NP_543136.1:p.Trp94Ter | rs587777635 | SNV | Stop gained |
|  | 8 | 21 | NM_080860.2:c.85G>T | NP_543136.1:p.Glu29Ter | rs138320978 | SNV | Stop gained |

Legend: N, number of alleles; chr, chromosome; HGVSc, Human Genome Variation Society coding sequence name; HGVSp, HGVS protein sequence name; dbSNP ID, Single Nucleotide Polymorphism database identification; SNV, single nucleotide variant; UTR, untranslated region; NA, not applicable; -, information is not available in the literature; *ARMC4*, Armadillo Repeat Containing 4; *CCDC151*, Coiled-Coil Domain Containing 151; *CCDC39*, Coiled-Coil Domain Containing 39; *CCDC40*, Coiled-Coil Domain Containing 40; *CFTR*, Cystic Fibrosis Transmembrane Conductance Regulator; *DNAAF3*, Dynein Axonemal Assembly Factor 3; *DNA11*, Dynein Axonemal Heavy Chain 11; *DNAH5*, Dynein Axonemal Heavy Chain 5; *DNAI2*, Dynein Axonemal Intermediate Chain 2; *DYX1C1-CCPG1*, Dyslexia Susceptibility 1 Candidate 1 and Cell Cycle Progression 1; *HYDIN*, Axonemal Central Pair Apparatus Protein; *RSPH1*, Radial Spoke Head 1 Homolog. ^a^, sift as deleterious (0.01) and PolyPhen as possibly damaging (0.776); b, sift as deleterious (0.02) and PolyPhen as benign (0.009).

Table S4. Complete information regarding the genetic variants with uncertain significance to pathogenicity screened in patients with phenotypes compatible with primary ciliary dyskinesia.

| Gene | N | Chr | HGVSc | HGVSp | dbSNP ID | Type | Consequence | Sift | PolyPhen |
| --- | --- | --- | --- | --- | --- | --- | --- | --- | --- |
| *ARMC4* | 1 | 10 | NM_018076.2:c.1813G>A | NP_060546.2:p.Val605Met | - | SNV | Missense variant | Tolerated (0.1) | Benign (0.079) |
|  | 1 | 10 | NM_018076.2:c.1592T>G | NP_060546.2:p.Ile531Ser | rs370164015 | SNV | Missense variant | Deleterious (0) | Probably damaging (0.992) |
|  | 1 | 10 | NM_018076.2:c.2408T>C | NP_060546.2:p.Val803Ala | rs757057849 | SNV | Missense variant | Deleterious (0.03) | Probably damaging (0.997) |
| *CCDC114* | 2 | 19 | NM_144577.3:c.1604C>G | NP_653178.3:p.Thr535Ser | - | SNV | Missense variant | Tolerated (0.44) | Benign (0.01) |
|  | 2 | 19 | NM_144577.3:c.455G>A | NP_653178.3:p.Arg152His | rs569554228 | SNV | Missense variant | Tolerated (0.07) | Benign (0.256) |
| *CCDC39* | 1 | 3 | NM_181426.1:c.1073C>T | NP_852091.1:p.Thr358Ile | rs183413880 | SNV | Missense variant | Deleterious (0.03) | Benign (0.113) |
|  | 1 | 3 | NM_181426.1:c.233G>A | NP_852091.1:p.Arg78His | rs11952495 | SNV | Missense variant | Tolerated (0.45) | Benign (0.009) |
|  | 2 | 3 | NM_181426.1:c.1781C>T | NP_852091.1:p.Thr594Ile | rs140505857 | SNV | Missense variant | Deleterious (0.01) | Possibly damaging (0.568) |
| *CCDC40* | 1 | 17 | NM_017950.3:c.1479G>T | NP_060420.2:p.Arg493Ser | rs201739201 | SNV | Missense variant | Deleterious (0) | Benign (0.23) |
|  | 1 | 17 | NM_017950.3:c.2267T>C | - | - | SNV | Missense variant | Deleterious (0.03) | Benign (0.347) |
|  | 1 | 17 | NM_017950.3:c.2900G>T | NP_060420.2:p.Arg967Leu | rs61686936 | SNV | Missense variant | Deleterious (0.02) | Benign (0.414) |
|  | 1 | 17 | NM_017950.3:c.2584C>T | NP_060420.2:p.Arg862Trp | rs201858385 | SNV | Missense variant | Deleterious (0.01) | Possibly damaging (0.497) |
| *CCDC65* | 1 | 12 | NM_033124.4:c.203T>C | NP_149115.2:p.Val68Ala | rs118060953 | SNV | Missense variant | Tolerated (0.16) | Benign (0.081) |
| *CFTR* | 1 | 7 | NM_000492.3:c.3705T>G | NP_000483.3:p.Ser1235Arg | rs34911792 | SNV | Missense variant | Tolerated (0.26) | Benign (0) |
|  | 3 | 7 | NM_000492.3:c.2002C>T | NP_000483.3:p.Arg668Cys | rs1800100 | SNV | Missense variant | Deleterious (0) | Probably damaging (0.96) |
| *DNAAF1* | 3 | 16 | NM_178452.4:c.1303G>A | NP_848547.4:p.Asp435Asn | rs149158199 | SNV | Missense variant | Tolerated (0.22) | Possibly damaging (0.658) |
|  | 1 | 16 | NM_178452.4:c.1161C>G | NP_848547.4:p.Asp387Glu | rs36062234 | SNV | Missense variant | Deleterious (0.03) | Possibly damaging (0.801) |
|  | 1 | 16 | NM_178452.4:c.*18T>C | - | rs2288024 | SNV | 3'-UTR |  |  |
| *DNAAF3* | 1 | 19 | NM_001256714.1:c.863G>T | NP_001243643.1:p.Arg288Leu | rs768515679 | SNV | Missense variant | Tolerated (0.06) | Benign (0.357) |
|  | 1 | 19 | NM_001256714.1:c.1555G>C | NP_001243643.1:p.Ala519Pro | rs546293010 | SNV | Missense variant | Deleterious (0) | Probably damaging (0.985) |
|  | 1 | 19 | NM_001256714.1:c.823G>A | NP_001243643.1:p.Gly275Ser | - | SNV | Missense variant | Deleterious (0) | Probably damaging (1) |
| *DNAH1* | 2 | 3 | NM_015512.4:c.1912G>A | NP_056327.4:p.Asp638Asn | rs201988957 | SNV | Missense variant |  | Benign (0.002) |
|  | 3 | 3 | NM_015512.4:c.1321G>C | NP_056327.4:p.Val441Leu | rs13060192 | SNV | Missense variant |  | Benign (0.027) |
|  | 1 | 3 | NM_015512.4:c.248C>T | NP_056327.4:p.Thr83Ile | rs775122473 | SNV | Missense variant |  | Benign (0.101) |
|  | 1 | 3 | NM_015512.4:c.6313C>G | NP_056327.4:p.Pro2105Ala | rs780811972 | SNV | Missense variant |  | Benign (0.103) |
|  | 1 | 3 | NM_015512.4:c.12098G>A | NP_056327.4:p.Arg4033Gln | rs762927513 | SNV | Missense variant |  | Benign (0.149) |
|  | 3 | 3 | NM_015512.4:c.11230C>T | NP_056327.4:p.Arg3744Cys | rs419752 | SNV | Missense variant |  | Possibly damaging (0.564) |
|  | 2 | 3 | NM_015512.4:c.4531G>A | NP_056327.4:p.Val1511Met | rs61734638 | SNV | Missense variant |  | Probably damaging (0.918) |
|  | 2 | 3 | NM_015512.4:c.4504G>A | NP_056327.4:p.Val1502Met | rs17052095 | SNV | Missense variant |  | Probably damaging (0.994) |
|  | 1 | 3 | NM_015512.4:c.9121C>T | NP_056327.4:p.Arg3041Cys | rs61731638 | SNV | Missense variant | Deleterious (0) | Probably damaging (0.998) |
|  | 1 | 3 | NM_015512.4:c.962_964delAGA | - | - | Deletion | In-frame deletion |  |  |
| *DNAH11* | 2 | 7 | NM_001277115.1:c.8023A>G | NP_001264044.1:p.Ile2675Val | rs72657364 | SNV | Missense variant | Tolerated (1) | Benign (0.001) |
|  | 3 | 7 | NM_001277115.1:c.2380G>A | NP_001264044.1:p.Glu794Lys | rs78653098 | SNV | Missense variant | Tolerated (0.79) | Benign (0.005) |
|  | 1 | 7 | NM_001277115.1:c.1535T>A | NP_001264044.1:p.Met512Lys | rs72655984 | SNV | Missense variant | Tolerated (0.52) | Benign (0.039) |
|  | 2 | 7 | NM_001277115.1:c.11233G>A | NP_001264044.1:p.Glu3745Lys | rs182389910 | SNV | Missense variant | Tolerated (0.31) | Benign (0.058) |
|  | 1 | 7 | NM_001277115.1:c.4124G>A | NP_001264044.1:p.Arg1375His | rs151018293 | SNV | Missense variant | Deleterious (0.03) | Benign (0.11) |
|  | 1 | 7 | NM_001277115.1:c.9097A>G | NP_001264044.1:p.Ile3033Val | rs72657373 | SNV | Missense variant | Tolerated (0.18) | Benign (0.298) |
|  | 1 | 7 | NM_001277115.1:c.9154G>A | NP_001264044.1:p.Val3052Ile | rs758713187 | SNV | Missense variant | Deleterious (0) | Benign (0.309) |
|  | 1 | 7 | NM_001277115.1:c.9241T>C | NP_001264044.1:p.Phe3081Leu | rs759593113 | SNV | Missense variant | Deleterious (0.01) | Benign (0.373) |
|  | 1 | 7 | NM_001277115.1:c.8990G>A | NP_001264044.1:p.Arg2997Gln | rs35865357 | SNV | Missense variant | Deleterious (0.01) | Possibly damaging (0.897) |
|  | 2 | 7 | NM_001277115.1:c.12561T>G | NP_001264044.1:p.Asp4187Glu | rs148429731 | SNV | Missense variant | Deleterious (0.02) | Probably damaging (0.928) |
|  | 2 | 7 | NM_001277115.1:c.5584A>G | NP_001264044.1:p.Asn1862Asp | rs769076802 | SNV | Missense variant | Deleterious (0) | Probably damaging (0.999) |
|  | 1 | 7 | NM_001277115.1:c.6019G>C | NP_001264044.1:p.Glu2007Gln | rs777145196 | SNV | Missense variant | Deleterious (0) | Probably damaging (0.999) |
| *DNAH11* | 2 | 7 | NM_001277115.1:c.13310G>A | NP_001264044.1:p.Arg4437His | rs775606157 | SNV | Missense variant | Deleterious (0) | Possibly damaging (0.832) |
| *DNAH5* | 1 | 5 | NM_001369.2:c.1858C>A | NP_001360.1:p.Gln620Lys | rs34076967 | SNV | Missense variant |  | Benign (0.14) |
|  | 1 | 5 | NM_001369.2:c.6632A>G | NP_001360.1:p.Asn2211Ser | rs561280268 | SNV | Missense variant |  | Benign (0.292) |
|  | 3 | 5 | NM_001369.2:c.7786A>G | NP_001360.1:p.Lys2596Glu |  | SNV | Missense variant |  | Possibly damaging (0.621) |
|  | 1 | 5 | NM_001369.2:c.2253C>A | NP_001360.1:p.Asn751Lys | rs115004914 | SNV | Missense variant |  | Possibly damaging (0.637) |
|  | 2 | 5 | NM_001369.2:c.4255T>C | NP_001360.1:p.Tyr1419His | rs143328219 | SNV | Missense variant |  | Possibly damaging (0.727) |
|  | 2 | 5 | NM_001369.2:c.12728G>A | NP_001360.1:p.Arg4243His | rs774987180 | SNV | Missense variant |  | Possibly damaging (0.812) |
|  | 1 | 5 | NM_001369.2:c.6235C>T | NP_001360.1:p.Leu2079Phe | rs371463953 | SNV | Missense variant |  | Possibly damaging (0.849) |
|  | 3 | 5 | NM_001369.2:c.11571-1G>A |  |  | SNV | Splice acceptor variant |  |  |
| *DNAH8* | 1 | 6 | NM_001206927.1:c.2980A>T | NP_001193856.1:p.Ile994Leu | rs141818413 | SNV | Missense variant |  | Benign (0.003) |
|  | 1 | 6 | NM_001206927.1:c.3215G>A | NP_001193856.1:p.Arg1072Gln | rs201654193 | SNV | Missense variant |  | Benign (0.041) |
|  | 2 | 6 | NM_001206927.1:c.10795G>A | NP_001193856.1:p.Val3599Ile | rs150171166 | SNV | Missense variant |  | Possibly damaging (0.679) |
|  | 1 | 6 | NM_001206927.1:c.11695C>T | NP_001193856.1:p.Arg3899Trp | rs138030174 | SNV | Missense variant |  | Probably damaging (1) |
| *DNAI1* | 1 | 9 | NM_001281428.1:c.1228G>A | NP_001268357.1:p.Gly410Ser | rs769177784 | SNV | Missense variant | Deleterious (0) | Probably damaging (0.997) |
| *DNAL1* | 2 | 14 | NM_031427.3:c.415C>G | NP_113615.2:p.Leu139Val | rs141873943 | SNV | Missense variant | Tolerated (0.09) | Benign (0.172) |
| *DRC1* | 1 | 2 | NM_145038.2:c.421T>G | NP_659475.2:p.Trp141Gly | rs139583194 | SNV | Missense variant | Deleterious (0) | Probably damaging (1) |
|  | 1 | 2 | NM_145038.2:c.1974C>G | NP_659475.2:p.Asp658Glu | rs140913454 | SNV | Missense variant | Deleterious (0.01) | Probably damaging (0.999) |
|  | 1 | 2 | NM_145038.2:c.*45G>A | - | rs11886898 | SNV | 3'-UTR |  |  |
| *DYX1C1*-  *]CCPG1* | 6 | 15 | NM_130810.3:c.988C>T | NP_570722.2:p.Arg330Trp | rs201173498 | SNV | Missense variant | Deleterious (0) | Probably damaging (1) |
|  | 1 | 15 | NM_130810.3:c.-13C>T | - | rs61753409 | SNV | 5'-UTR |  |  |
|  | 1 | 15 | NM_130810.3:c.1249G>T | NP_570722.2:p.Glu417Ter | rs57809907 | SNV | Stop gained |  |  |
| *HYDIN* | 1 | 16 | NM_001270974.1:c.8063T>A | NP_001257903.1:p.Ile2688Lys | rs77324634 | SNV | Missense variant |  | Benign (0.008) |
|  | 3 | 16 | NM_001270974.1:c.7588A>G | NP_001257903.1:p.Lys2530Glu | rs1798528 | SNV | Missense variant |  | Benign (0.018) |
|  | 1 | 16 | NM_001270974.1:c.9409C>A | NP_001257903.1:p.Arg3137Ser | rs369447981 | SNV | Missense variant |  | Benign (0.021) |
|  | 1 | 16 | NM_001270974.1:c.9913G>A | NP_001257903.1:p.Gly3305Ser | rs185616885 | SNV | Missense variant |  | Benign (0.023) |
|  | 1 | 16 | NM_001270974.1:c.11495G>A | NP_001257903.1:p.Arg3832His | rs7198721 | SNV | Missense variant |  | Benign (0.038) |
|  | 1 | 16 | NM_001270974.1:c.12417G>A | NP_001257903.1:p.Met4139Ile | rs374719281 | SNV | Missense variant |  | Benign (0.216) |
|  | 1 | 16 | NM_001270974.1:c.8674C>G | NP_001257903.1:p.Gln2892Glu | - | SNV | Missense variant,  splice region variant |  | Benign (0.388) |
|  | 1 | 16 | NM_001270974.1:c.12808C>T | NP_001257903.1:p.His4270Tyr | rs111318087 | SNV | Missense variant |  | Possibly damaging (0.607) |
|  | 1 | 16 | NM_001270974.1:c.97A>G | NP_001257903.1:p.Ser33Gly | rs75357093 | SNV | Missense variant |  | Possibly damaging (0.714) |
|  | 1 | 16 | NM_001270974.1:c.1466G>A | NP_001257903.1:p.Gly489Asp | rs62040318 | SNV | Missense variant |  | Possibly damaging (0.725) |
|  | 2 | 16 | NM_001270974.1:c.12263A>G | NP_001257903.1:p.Lys4088Arg | rs1774416 | SNV | Missense variant |  | Possibly damaging (0.801) |
|  | 3 | 16 | NM_001270974.1:c.12076G>A | NP_001257903.1:p.Ala4026Thr | rs11075798 | SNV | Missense variant |  | Possibly damaging (0.908) |
|  | 1 | 16 | NM_032821.2:c.15355A>G | NP_116210.2:p.Thr5119Ala | - | SNV | Missense variant | Tolerated (0.08) | Possibly damaging (0.712) |
|  | 1 | 16 | NM_001270974.1:c.3374A>T | NP_001257903.1:p.Lys1125Met | rs79123258 | SNV | Missense variant |  | Probably damaging (0.923) |
|  | 2 | 16 | NM_001270974.1:c.11695G>A | NP_001257903.1:p.Val3899Met | rs1626593 | SNV | Missense variant |  | Probably damaging (0.938) |
|  | 3 | 16 | NM_001270974.1:c.11242C>T | NP_001257903.1:p.Arg3748Cys | rs12102644 | SNV | Missense variant |  | Probably damaging (0.942) |
|  | 1 | 16 | NM_001270974.1:c.5627G>A | NP_001257903.1:p.Arg1876Gln | rs371318679 | SNV | Missense variant |  | Probably damaging (0.947) |
|  | 2 | 16 | NM_001270974.1:c.8795C>T | NP_001257903.1:p.Pro2932Leu | rs11075812 | SNV | Missense variant |  | Probably damaging (0.96) |
|  | 1 | 16 | NM_001270974.1:c.4463A>G | NP_001257903.1:p.Glu1488Gly | rs201819005 | SNV | Missense variant |  | Probably damaging (0.969) |
|  | 1 | 16 | NM_001270974.1:c.5152G>A | NP_001257903.1:p.Val1718Met | rs783762 | SNV | Missense variant |  | Probably damaging (0.986) |
|  | 1 | 16 | NM_001270974.1:c.6157G>C | NP_001257903.1:p.Ala2053Pro | - | SNV | Missense variant |  | Probably damaging (0.991) |
|  | 1 | 16 | NM_001270974.1:c.-151G>T | - | rs115761180 | SNV | 5'-UTR |  |  |
|  | 1 | 16 | NM_001270974.1:c.7561_7575  delACGGAGAAGGAGCGC | NP_001257903.1:p.Thr2521_  Arg2525del | rs375727122;  rs67115747 | Deletion | In-frame deletion |  |  |
|  | 1 | 16 | NM_001270974.1:c.3043-1G>A | - | - | SNV | Splice acceptor variant |  |  |
|  | 1 | 16 | NM_001270974.1:c.12295+1G>A | - | - | SNV | Splice donor variant |  |  |
| *NA* | 1 | 19 | NM_001256714.1:c.-44C>T | - | rs73066642 | SNV | 5'-UTR |  |  |
| *RPGR* | 1 | X | NM_001034853.1:c.3074_3085  delTGGAAGGGGAGG | NP_001030025.1:p.Val1025_  Glu1028del | rs201134185 | Deletion | In-frame deletion |  |  |
| *RSPH4A* | 1 | 6 | NM_001010892.2:c.1489G>A | NP_001010892.1:p.Val497Ile | rs117169123 | SNV | Missense variant | Tolerated (0.55) | Benign (0.176) |
|  | 1 | 6 | NM_001010892.2:c.731G>A | NP_001010892.1:p.Arg244His | rs41289942 | SNV | Missense variant | Deleterious (0.05) | Benign (0.284) |
| *TTC14*,  *CCDC39* | 1 | 3 | NM_181426.1:c.2640A>T | NP_852091.1:p.Arg880Ser | - | SNV | Missense variant | Tolerated (0.3) | Benign (0.059) |
| *ZMYND10* | 1 | 3 | NM_015896.2:c.*39G>A | - | rs78166579 | SNV | 3'-UTR |  |  |

Legend: N, number of alleles; chr, chromosome; HGVSc, Human Genome Variation Society coding sequence name; HGVSp, HGVS protein sequence name; dbSNP ID, Single Nucleotide Polymorphism database identification; SNV, single nucleotide variant; UTR, untranslated region; NA, not applicable; -, information is not available in the literature; *ARMC4*, Armadillo Repeat Containing 4; *CCDC114*, Coiled-Coil Domain Containing 114; *CCDC39*, Coiled-Coil Domain Containing 39; *CCDC40*, Coiled-Coil Domain Containing 40; *CCDC65*, Coiled-Coil Domain Containing 65; *CFTR*, Cystic Fibrosis Transmembrane Conductance Regulator; *DNAAF1*, Dynein Axonemal Assembly Factor 1; *DNAAF3*, Dynein Axonemal Assembly Factor 3; *DNAH1*, Dynein Axonemal Heavy Chain 1; *DNA11*, Dynein Axonemal Heavy Chain 11; *DNAH5*, Dynein Axonemal Heavy Chain 5; *DNAH8*, Dynein Axonemal Heavy Chain 8; *DNAI1*, Dynein Axonemal Intermediate Chain 1; *DNAL1*, Dynein Axonemal Light Chain 1; *DRC1*, Dynein Regulatory Complex Subunit 1; *DYX1C1-CCPG1*, Dyslexia Susceptibility 1 Candidate 1 and Cell Cycle Progression 1; *HYDIN*, Axonemal Central Pair Apparatus Protein; *RPGR*, Retinitis Pigmentosa Gtpase Regulator; *RSPH4A*, Radial Spoke Head 4 Homolog A; *TTC14*, Tetratricopeptide Repeat Domain 14; *ZMYND10*, Zinc Finger, MYND-type Containing 10.

Table S5**.** Related genotypes screened in patients with phenotypes compatible with primary ciliary dyskinesia.

| Patient^§^ | Allele 1 | Allele 2 | Uncertain significance |
| --- | --- | --- | --- |
| Br-39 | No | No | (*DYX1C1-CCPG1*) NM_130810.3:c.988C>T;  (*HYDIN*) NM_001270974.1:c.9913G>A |
| Br-34  and Br-13 | No | No | (*DNAH5*) NM_001369.2:c.12728G>A |
| Br-5, Br-31  and Br-30 | No | No | (*CFTR*)** NM_000492.3:c.2002C>T;  (*DNAH5*) NM_001369.2:c.11571-1G>A, NM_001369.2:c.7786A>G |
| Br-40  and Br-41 | No | No | (*CCDC40*)* NM_017950.3:c.2584C>T;  (*DNAH11*) NM_001277115.1:c.8023A>G;  (*DNAL1*) NM_031427.3:c.415C>G;  (*DYX1C1-CCPG1*) NM_130810.3:c.988C>T |
| Br-43 | No | No | (*DNAH1*) NM_015512.4:c.1912G>A, NM_015512.4:c.4531G>A;  (*DNAH5*) NM_001369.2:c.2253C>A |
| Br-6 | No | No | (*CCDC39*) NM_181426.1:c.1073C>T;  (*RPGR*)*** NM_001034853.1:c.3074_3085delTGGAAGGGGAGG |
| Br-47 | No | No | No |
| Br-48 | No | No | (*DNAH1*) NM_015512.4:c.1321G>C, NM_015512.4:c.11230C>T;  (*DNAH11*) NM_001277115.1:c.1535T>A; NM_001277115.1:c.9154G>A;  (*HYDIN*) NM_001270974.1:c.5152G>A |
| Br-42 | No | No | (*CFTR*) NM_000492.3:c.2002C>T;  (*DNAAF1)* NM_178452.4:c.*18T>C;  (*DNAAF3*) NM_001256714.1:c.863G>T;  (*DNAH1*) NM_015512.4:c.12098G>A;  (*DNAH8*) NM_001206927.1:c.3215G>A;  (*HYDIN*) NM_001270974.1:c.12295+1G>A |
| Br-1 | No | No | (*DNAI1*) NM_001281428.1:c.1228G>A;  (*HYDIN*) NM_001270974.1:c.12263A>G |
| Br-9 | No | No | (*CCDC40*) NM_017950.3:c.2900G>T;  (*HYDIN*) NM_001270974.1:c.4463A>G, NM_001270974.1:c.9409C>A,  NM_001270974.1:c.5627G>A, NM_001270974.1:c.12417G>A, NM_001270974.1:c.11495G>A, NM_001270974.1:c.8063T>A |
| Br-22 | No | No | (*DNAFF3*) NM_001256714.1:c.1555G>C;  (*CCDC39*) NM_181426.1:c.233G>A |
| Br-4 | (*CFTR*) NM_000492.3:c.350G>A | No | (*DNAH11*) NM_001277115.1:c.9241T>C, NM_001277115.1:c.6019G>C;  (*DYX1C1-CCPG1*) NM_130810.3:c.1249G>T; NM_130810.3:c.-13C>T |
| Br-45 | (*RSPH1*) NM_080860.2:c.85G>T | No | (*HYDIN*) NM_001270974.1:c.7588A>G, NM_001270974.1:c.6157G>C, NM_001270974.1:c.3043-1G>A, NM_001270974.1:c.9913G>A |
| Br-19, Br-15  and Br-20 | (*DYX1C1-CCPG1*) NM_130810.3:c.862_866delAAGAA | No | (*CFTR*)* NM_000492.3:c.3705T>G;  (*DNAH11*)^#,^* NM_001277115.1:c.2380G>A;  (*DNAH5*) NM_001369.2:c.4255T>C |
| BR- 49 | (*ARMC4*) NM_018076.2:c.97G>T | No | (*ARMC4*) NM_018076.2:c.1813G>A, NM_018076.2:c.1592T>G;  (*DNAH1*) NM_015512.4:c.6313C>G;  (*DRC1*) NM_145038.2:c.421T>G |
| Br-46 | (*HYDIN*) NM_001270974.1:c.11617dupA | No | *DNAH11* (NM_001277115.1:c.11233G>A) |
| Br-44 | (*HYDIN*) NM_001270974.1:c.8675delA | No | (*DNAH1*) NM_015512.4:c.1912G>A, NM_015512.4:c.4531G>A;  (*HYDIN*) NM_001270974.1:c.8674C>G, NM_001270974.1:c.12076G>A,  NM_001270974.1:c.11695G>A, NM_001270974.1:c.7588A>G |
| Br-32  and Br-28 | (*CCDC40*) NM_017950.3:c.1989+1G>A | (*CCDC40*) NM_017950.3:c.1989+1G>A | (*CCDC114*) NM_144577.3:c.1604C>G, NM_144577.3:c.455G>A;  (*CCDC39*) NM_181426.1:c.1781C>T;  (*DNAH11*) NM_001277115.1:c.5584A>G;  (*DNAH11*, *CDCA7L*) NM_001277115.1:c.13310G>A;  (*DNAH8*) NM_001206927.1:c.10795G>A;  (*HYDIN*)* NM_001270974.1:c.12076G>A |
| Br-24 | (*DNAH5*) NM_001369.2:c.4237C>T | (*DNAH5*) NM_001369.2:c.4237C>T | (*CCDC40*) NM_017950.3:c.2267T>C;  (*CCDC65*) NM_033124.4:c.203T>C;  (*DNAAF1*) NM_178452.4:c.1303G>A;  (*DRC1*) NM_145038.2:c.*45G>A;  (*HYDIN*) NM_001270974.1:c.-151G>T;  (*ZMYND10*) NM_015896.2:c.*39G>A; NM_001256714.1:c.-44C>T |
| Br-3 | (*DNAI2*) NM_023036.4:c.787C>T | (*DNAI2*) NM_023036.4:c.787C>T | (*HYDIN*) NM_001270974.1:c.8795C>T, NM_001270974.1:c.12808C>T,  NM_001270974.1:c.11242C>T;  (*TTC14, CCDC39*) NM_181426.1:c.2640A>T |
| Br-10 | (*CCDC39*) NM_181426.1:c.2497_2498delCA) | (*CCDC39*) NM_181426.1:c.2497_2498  delCA) | (*DNAAF1*) NM_178452.4:c.1161C>G;  (*DNAH1*) NM_015512.4:c.9121C>T;  (*DNAH11*) NM_003777.3:c.12583T>G;  (*HYDIN*) NM_032821.2:c.15355A>G |
| Br-16 | (*CCDC40*) NM_017950.3:c.3097A>T | (*CCDC40*) NM_017950.3:c.3097A>T | (*DNAAF1*) NM_178452.4:c.1303G>A;  (*DRC1*) NM_145038.2:c.1974C>G |
| Br-23  and Br-29 | (*RSPH1*) NM_080860.2:c.85G>T | (*RSPH1*) NM_080860.2:c.85G>T | No |
| Br-21 | (*DNAH5*) NM_001369.2:c.13486C>T | (*DNAH5*) NM_001369.2:c.13486C>T | (*ARMC4*) NM_018076.2:c.2408T>C |
| Br-12 | (*CCDC151*) NM_145045.4:c.583_595  dupGCGCAAAACAGAC | (*CCDC151*) NM_145045.4:c.583_595  dupGCGCAAAACAGAC | (*CCDC40*) NM_017950.3:c.1479G>T;  (*DNAH5*) NM_001369.2:c.6235C>T, NM_001369.2:c.6632A>G;  (*DNAH8*) NM_001206927.1:c.11695C>T |
| Br-7 | (*CCDC40*) NM_017950.3:c.2920C>T | (*CCDC40*) NM_017950.3:c.2920C>T | No |
| Br-11  and Br-25 | (*CCDC40*) NM_017950.3:c.1571delA | (*CCDC40*) NM_017950.3:c.1571delA | (*HYDIN*)*: NM_001270974.1:c.12076G>A, NM_001270974.1:c.11695G>A,  NM_001270974.1:c.12263A>G, NM_001270974.1:c.3374A>T;  (*DNAH11*)* NM_001277115.1:c.11233G>A;  (*RSPH4A*)* NM_001010892.2:c.1489G>A |
| Br-14 | (*DNAAF3*) NM_001256714.1:c.1201dupG | (*DNAAF3*) NM_001256714.1:c.1201dupG | (*DNAAF3*) NM_001256714.1:c.823G>A;  (*HYDIN*) NM_001270974.1:c.97A>G;  (*RSPH4A*) NM_001010892.2:c.731G>A |
| Br-8 | (*CCDC40*) NM_017950.3: c.1232dupT;  (*DNAH11*) NM_001277115.1: c.8533C>G | (*CCDC40*) NM_017950.3:c.1232dupT | (*DNAH1*) NM_015512.4: c.4504G>A;  (*DNAH5*) NM_001369.2:c.1858C>A;  (*HYDIN*) NM_001270974.1:c.8795C>T, NM_001270974.1:c.11242C>T |
| Br-35 | (*CCDC40*) NM_017950.3:c.1571delA | (*CCDC40*) NM_017950.3:c.1571delA | (*HYDIN*) NM_001270974.1:c.7561_7575delACGGAGAAGGAGCGC,  NM_001270974.1:c.1466G>A |
| Br-17 | (*RSPH1*) NM_080860.2:c.85G>T | (*RSPH1*) NM_080860.2:c.85G>T | (*DNAH1*) NM_015512.4:c.962_964delAGA;  (*DNAH11*) NM_001277115.1:c.4124G>A |
| Br-18 | (*DNAH5*) NM_001369.2:c.11653C>T | (*DNAH5*) NM_001369.2:c.13458dupT | No |
| Br-26  and Br-27 | (*DNAH5*) NM_001369.2:c.13060delG | (*DNAH5*) NM_001369.2:c.13060delG | (*DNAH1*)* NM_015512.4:c.4504G>A;  (*DNAH11*)* NM_015512.4:c.1321G>C, NM_015512.4:c.11230C>T,  NM_015512.4:c.248C>T, NM_001277115.1:c.8990G>A |
| Br-37 | (*RSPH1*) NM_080860.2:c.85G>T | (*RSPH1*) NM_080860.2:c.275-2A>C | (*DNAAF1*) NM_178452.4:c.1303G>A;  (*DNAH1*) NM_015512.4:c.1321G>C, NM_015512.4:c.11230C>T;  (*DNAH11*) NM_001277115.1:c.9097A>G |
| Br-2 | (*RSPH1*) NM_080860.2:c.275-2A>C | (*RSPH1*) NM_080860.2:c.281G>A | (*DNAH11*) NM_001277115.1:c.12561T>G;  (*DNAH8*) NM_001206927.1:c.2980A>T;  (*HYDIN*) NM_001270974.1:c.11242C>T, NM_001270974.1:c.7588A>G |
| Br-33 | (*DNAH5*) NM_001369.2:c.2090T>A | (*DNAH5*) NM_001369.2:c.13486C>T | No |

Legend: *ARMC4*, Armadillo Repeat Containing 4; *CCDC151*, Coiled-Coil Domain Containing 151; *CCDC114*, Coiled-Coil Domain Containing 114; *CCDC39*, Coiled-Coil Domain Containing 39; *CCDC40*, Coiled-Coil Domain Containing 40; *CCDC65*, Coiled-Coil Domain Containing 65; *CFTR*, Cystic Fibrosis Transmembrane Conductance Regulator; *DNAAF1*, Dynein Axonemal Assembly Factor 1; *DNAAF3*, Dynein Axonemal Assembly Factor 3; *DNAH1*, Dynein Axonemal Heavy Chain 1; *DNA11*, Dynein Axonemal Heavy Chain 11; *CDCA7L*, Cell Division Cycle Associated 7 Like; *DNAH5*, Dynein Axonemal Heavy Chain 5; *DNAH8*, Dynein Axonemal Heavy Chain 8; *DNAI1*, Dynein Axonemal Intermediate Chain 1; *DNAI1*, Dynein Axonemal Intermediate Chain 1; *DNAI2*, Dynein Axonemal Intermediate Chain 2; *DNAL1*, Dynein Axonemal Light Chain 1; *DRC1*, Dynein Regulatory Complex Subunit 1; *DYX1C1-CCPG1*, Dyslexia Susceptibility 1 Candidate 1 and Cell Cycle Progression 1; *HYDIN*, Axonemal Central Pair Apparatus Protein; *RPGR*, Retinitis Pigmentosa GTPase Regulator; *RSPH1*, Radial Spoke Head 1 Homolog; *RSPH4A*, Radial Spoke Head 4 Homolog A; *TTC14*, Tetratricopeptide Repeat Domain 14; *ZMYND10*, Zinc Finger, MYND-type Containing 10. *, occurred in one patient; **, occurred in two patients; ***, the variant was screened in the X chromosome in a male patient; ^#^, one patient was homozygous; **^§^**, in cases of siblings, more than 1 participant is shown on the same line. The bold type represents homozygous.

REFERENCES

Bibliography

1. Harris, A. *et al.* Validation of portable nitric oxide analyzer for screening in primary ciliary dyskinesias. *BMC Pulm Med* **14,** 1–8 (2014).

2. Marthin, J. K. & Nielsen, K. G. Hand-held tidal breathing nasal nitric oxide measurement--a promising targeted case-finding tool for the diagnosis of primary ciliary dyskinesia. *PLoS One* **8,** e57262 (2013).

3. Montella, S. *et al.* Measurement of nasal nitric oxide by hand-held and stationary devices. *Eur. J. Clin. Invest.* **41,** 1063–1070 (2011).

4. Olm, M. A. K. *et al.* Primary ciliary dyskinesia: evaluation using cilia beat frequency assessment via spectral analysis of digital microscopy images. *J. Appl. Physiol.* **111,** 295–302 (2011).

5. Shoemark, A., Dixon, M., Corrin, B. & Dewar, A. Twenty-year review of quantitative transmission electron microscopy for the diagnosis of primary ciliary dyskinesia. *J Clin Pathol.* **65,** 267–271 (2012).

6. Carlén, B. & Stenram, U. Primary ciliary dyskinesia: a review. *Ultrastruct Pathol* **29,** 217–20 (2005).

7. Roomans, G. M., Ivanovs, A., Shebani, E. B. & Johannesson, M. Transmission electron microscopy in the diagnosis of primary ciliary dyskinesia. *Ups. J. Med. Sci.* **111,** 155–68 (2006).

8. Smith, T. F. & Waterman, M. S. Identification of common molecular subsequences. *J. Mol. Biol.* **147,** 195–7 (1981).

9. Robinson, J. T. *et al.* Integrative genomics viewer. *Nat. Biotechnol.* **29,** 24–6 (2011).

10. Thorvaldsdóttir, H., Robinson, J. T. & Mesirov, J. P. Integrative Genomics Viewer (IGV): high-performance genomics data visualization and exploration. *Brief. Bioinform.* **14,** 178–92 (2013).

11. Richards, S. *et al.* Standards and guidelines for the interpretation of sequence variants: a joint consensus recommendation of the American College of Medical Genetics and Genomics and the Association for Molecular Pathology. *Genet. Med.* **17,** 405–423 (2015).

12. Richards, S. *et al.* Standards and guidelines for the interpretation of sequence variants: a joint consensus recommendation of the American College of Medical Genetics and Genomics and the Association for Molecular Pathology. *Genet. Med.* **17,** 405–423 (2015).

13. Li, Q. & Wang, K. InterVar: Clinical Interpretation of Genetic Variants by the 2015 ACMG-AMP Guidelines. *Am. J. Hum. Genet.* **100,** 267–280 (2017).

14. Vaughan, J. *et al.* Exhaled breath condensate pH is a robust and reproducible assay of airway acidity. *Eur. Respir. J.* **22,** 889–94 (2003).

15. Goto, D. M. *et al.* Furosemide impairs nasal mucociliary clearance in humans. *Respir. Physiol. Neurobiol.* **170,** 246–52 (2010).
